# Striatal spatial heterogeneity, clustering, and white matter association of GFAP^+^ astrocytes in a mouse model of Huntington’s disease

**DOI:** 10.3389/fncel.2023.1094503

**Published:** 2023-04-28

**Authors:** Taylor G. Brown, Mackenzie N. Thayer, Jillian G. VanTreeck, Nicole Zarate, Damyan W. Hart, Sarah Heilbronner, Rocio Gomez-Pastor

**Affiliations:** Department of Neuroscience, School of Medicine, University of Minnesota, Minneapolis, MN, United States

**Keywords:** Huntington’s disease (HD), astrocytes, striatum, white matter (WM), glial fibrillary acidic protein (GFAP), S100B

## Abstract

**Introduction:**

Huntington’s disease (HD) is a neurodegenerative disease that primarily affects the striatum, a brain region that controls movement and some forms of cognition. Neuronal dysfunction and loss in HD is accompanied by increased astrocyte density and astrocyte pathology. Astrocytes are a heterogeneous population classified into multiple subtypes depending on the expression of different gene markers. Studying whether mutant Huntingtin (HTT) alters specific subtypes of astrocytes is necessary to understand their relative contribution to HD.

**Methods:**

Here, we studied whether astrocytes expressing two different markers; glial fibrillary acidic protein (GFAP), associated with astrocyte activation, and S100 calcium-binding protein B (S100B), a marker of matured astrocytes and inflammation, were differentially altered in HD.

**Results:**

First, we found three distinct populations in the striatum of WT and symptomatic zQ175 mice: GFAP^+^, S100B^+^, and dual GFAP^+^S100B^+^. The number of GFAP^+^ and S100B^+^ astrocytes throughout the striatum was increased in HD mice compared to WT, coinciding with an increase in HTT aggregation. Overlap between GFAP and S100B staining was expected, but dual GFAP^+^S100B^+^ astrocytes only accounted for less than 10% of all tested astrocytes and the number of GFAP^+^S100B^+^ astrocytes did not differ between WT and HD, suggesting that GFAP^+^ astrocytes and S100B^+^ astrocytes are distinct types of astrocytes. Interestingly, a spatial characterization of these astrocyte subtypes in HD mice showed that while S100B^+^ were homogeneously distributed throughout the striatum, GFAP^+^ preferentially accumulated in “patches” in the dorsomedial (dm) striatum, a region associated with goal-directed behaviors. In addition, GFAP^+^ astrocytes in the dm striatum of zQ175 mice showed increased clustering and association with white matter fascicles and were preferentially located in areas with low HTT aggregate load.

**Discussion:**

In summary, we showed that GFAP^+^ and S100B^+^ astrocyte subtypes are distinctly affected in HD and exist in distinct spatial arrangements that may offer new insights to the function of these specific astrocytes subtypes and their potential implications in HD pathology.

## Introduction

Huntington’s disease (HD) is a devastating neurodegenerative disease that manifests as progressive motor, cognitive, and psychiatric impairments (Roos, 2010). A CAG (glutamine) triplet expansion in the Huntingtin gene (*HTT*) produces a dysfunctional mutant protein (mHTT) that is prone to misfolding and aggregation (MacDonald et al., 1993). mHTT preferentially affects medium spiny neurons (MSNs) in the striatum and leads to severe degeneration of this brain region. Anatomical analyses showed that within the striatum the dorsal striatum is severely affected (Bates et al., 2015; Morigaki and Goto, 2017), although the reason for such region selectivity is uncertain since mHTT expression and aggregation is found throughout the brain.

Neuronal dysfunction and loss of MSNs in HD is also accompanied by increased astrocyte density and astrocyte pathology (Vonsattel et al., 1985; Verkhratsky et al., 2019). Astrocytes are glial cells with a variety of homeostatic, synaptic, and neuroprotective functions (Araque et al., 1999; Eroglu and Barres, 2010; Verkhratsky et al., 2019). Specifically, an increased density of astrocytes and an upregulation of the astrocytic glial fibrillary acidic protein (GFAP), considered a canonical marker of astrocyte reactivity (Buffo et al., 2008; Kamphuis et al., 2012; Hol and Pekny, 2015; Escartin et al., 2021), is seen in postmortem brains of patients with HD (Selkoe et al., 1982; Vonsattel et al., 1985). Additionally, astrocyte dysfunction, in terms of ion homeostasis, Ca^2+^ signaling, and neurotransmitter clearance, is a key factor in both the onset and progression of HD symptoms (Khakh et al., 2017; Diaz-Castro et al., 2019; Al-Dalahmah et al., 2020). When mHTT is explicitly expressed in mouse astrocytes there is a progressive disruption of astrocytic glutamate transport and the manifestation of some HD-like phenotypes (Faideau et al., 2010), while reduction of mHTT in astrocytes of BACHD mice partially improved neuronal excitability and motor behavior (Wood et al., 2019). Although these studies support a fundamental role of astrocytes in HD, it is still unclear whether all astrocytes within the striatum contribute similarly to HD.

Contrary to previous knowledge, recent transcriptomic studies have shown that astrocytes are a heterogeneous group of cells that differ transcriptionally and morphologically by brain region (Sosunov et al., 2014; Khakh and Deneen, 2019; Batiuk et al., 2020; Hasel et al., 2021; Ohlig et al., 2021) and can have multiple, distinct states of reactivity depending on the environmental perturbations (Liddelow et al., 2017; Al-Dalahmah et al., 2020; Yu et al., 2020; Hasel et al., 2021). Single cell RNA-seq (scRNA-seq) evidence in postmortem HD patient brains demonstrated distinct subtypes of astrocytes that are categorized based on the expression of different astrocytic markers and implied multiple response states in HD (Al-Dalahmah et al., 2020). Therefore, our previous understanding of the contribution of astrocytes to HD may be incomplete. These studies raised new questions as to whether different subtypes of astrocytes may be preferentially affected in HD and whether they play a differential role in HD pathology.

Given the clear evidence for astrocyte heterogeneity and their involvement in HD, we sought to determine whether different subtypes of astrocytes present different states of astrogliosis and whether they shared a common or distinct spatial distribution throughout the striatum. We studied two subtypes of astrocytes, each expressing a different astrocyte marker, S100 calcium binding protein B (S100B), a marker of matured astrocytes and inflammation, or GFAP, in the brain of wildtype (WT) and the heterozygous zQ175 (HD) mice. The zQ175 mouse model is an ideal model to study disease progression, as they exhibit a slow accumulation of HTT aggregates, striatal degeneration, and motor symptoms. We conducted immunofluorescence (IF) and microscopy analyses to assess number, spatial distribution, and clustering of different astrocytes subtypes. Our results demonstrate three distinct populations in the striatum of WT and symptomatic zQ175 mice: GFAP^+^, S100B^+^, and dual GFAP^+^S100B^+^. While GFAP^+^S100B^+^ only accounted for less than 10% of all tested astrocytes and they did not significantly differ between WT and HD, the abundance of GFAP^+^ and S100B^+^ astrocytes increased in HD mice compared to WT coinciding with increased HTT aggregation. We also found that while S100B^+^ were homogeneously distributed throughout the striatum, GFAP^+^ preferentially accumulated in the dorsomedial (dm) striatum. GFAP^+^ astrocytes in the dm striatum of zQ175 mice showed increased clustering and association with white matter fascicles and were preferentially located in areas with low HTT aggregate load. Overall, our data demonstrated a differential alteration in the number and spatial distribution of different astrocyte subtypes in the striatum of HD mice that could have a direct impact in understanding their distinct roles in pathology.

## Materials and methods

### Mouse lines

For this study we used a full-length knock-in mouse model of HD known as zQ175, which harbors a chimeric human/mouse exon 1 carrying an expansion of ∼165 CAG repeats and the human poly-proline region (Menalled et al., 2012; Yu et al., 2022). We chose this mouse model because it shows slow and progressive HTT aggregation, striatal degeneration, and motor dysfunction and better reproduces human genetics and disease pathology compared to transgenic mouse models (Menalled et al., 2012). WT (C57BL/6J) animals were used as controls. Both heterozygous zQ175 mice and WT mice were obtained from crosses of heterozygous zQ175 mice with WT mice. Mice selected from a total of 23 litters with an average of 7 pups per litter, including ages 3, 6, 12, and 18 months, were used. Sample size was set to a minimum of five animals per genotype for every analysis. When possible, a balanced number of males and females were used. When sample sizes allowed, potential sex differences were assessed. No randomization of animals was used in this study. All animal care and sacrifice procedures were approved by the University of Minnesota Institutional Animal Care and Use Committee (IACUC) in compliance with the National Institutes of Health guidelines for the care and use of laboratory animals under the approved animal protocol 2007-A38316.

### Immunohistochemistry

Mice were anesthetized with Avertin (250 mg/kg Tribromoethanol) and perfused intracardially with tris-buffered saline (TBS) (25 mM Tris-base, 135 mM Nacl, 3 mM KCl, pH 7.6) supplemented with 7.5 mM heparin. Brains were dissected, fixed with 4% PFA in TBS at 4°C for 4–5 days, cryoprotected with 30% sucrose in TBS for 4–5 days and embedded in a 2:1 mixture of 30% sucrose in TBS:OCT (Tissue-Tek), as previously described (Gomez-Pastor et al., 2017). Brains were cryo-sectioned into 16 μm-thick coronal (between +1.0 and +0.2 mm from Bregma) or sagittal sections (between 1.0 and 2.0 mm lateral of the midline), washed and permeabilized in TBS with 0.2% Triton X-100 (TBST). For each experiment, three sections at intervals of approximately 0.2 mm were used per mouse. Sections were blocked in 5% normal goat serum (NGS) in TBST for 1 h at room temperature. Primary antibodies were incubated overnight at 4°C in TBST containing 5% NGS. Secondary Alexa-fluorophore-conjugated antibodies (Invitrogen) were added (1:200 in TBST with 5% NGS) for 1 h at room temperature. Slides were mounted in ProLong Gold Antifade with DAPI (Invitrogen) and subsequently imaged. Primary antibodies used and dilutions are as follows: GFAP (Rabbit, Invitrogen PA1-10019, 1:500), GFAP (chicken, ab5541, 1:2000), S100B (Rabbit, Abcam ab41548, 1:500), EM48 (mouse, mab5374, 1:500), and MBP (rabbit, 10458-1-AP, 1:2000).

### Imaging and image analysis

Images were acquired on an epi-fluorescent microscope (Echo Revolve), a macro zoom system (MVX10, Olympus), or a confocal microscope (Stellaris, Leica). On the Echo Revolve, 1.25× images have a 0.4467 pixel/μm resolution, 10× images have a 3.569 pixel/μm resolution, and 20× images have a 7.1 pixel/μm resolution. All Echo images have a 2732 × 1948 pixel size. On the MVX-10, images have a 0.15 pixel/μm resolution and 1376 × 1038 pixel size. On the Stellaris, 10× tiled images have a 0.17 pixel/μm resolution, 512 × 512 pixel size, and a z-stack encompassing the entire 16 μm was acquired. Stellaris 20× images have a 0.88 pixel/μm resolution, 512 × 512 pixel size, and a z-stack encompassing the entire 16 μm was acquired. Images were taken of the entire slice for 1.25× images using the Echo Revolve and MVX-10 microscopes. For 10× and 20× images, three to four images were taken at consistent locations in the striatum corresponding to the dm, dl, cm, and cl, which was located using anatomical adjacent structures as a reference and DAPI staining. For each brain slice, one representative image was taken in each striatal subregion, and data obtained from three images corresponding to three independent brain slices were averaged. Images acquired on the Stellaris accommodated for a fourth image per slice in the dl striatum. To avoid overlapping, each image within the striatum was captured with at least 100 μm separation to image dm, dl, cm, and cl and images never overlapped. Images were analyzed in their entirety for cell counting. Image settings including exposure time, binning, and thresholding were set using images obtained from representative striatum area in WT animals and identical settings were applied to images captured in zQ175 mice.

#### Cell counting

For counting analyses, the cell counter plugin from ImageJ software was used and cells/fascicles were counted manually. For GFAP^+^ and S100B^+^ cell counting in 12-month-old mice, we used epifluorescent images with no z-dimension. For GFAP^+^ cell counting and fascicle counting in 18-month-old mice, we used confocal images which were acquired with a 16 μm z-dimension and condensed using the Z Project function on Image J at max intensity. Cells or fascicles on the border of images were counted. All images used to calculate whole striatum cell counts were taken sampling throughout the striatum using the same area per image (0.4 mm^2^) and cell counts were averaged from three independent images (each corresponding to dm, cm, and cl).

#### Fluorescence intensity analyses

For intensity analyses, we used epifluorescent images with no z-dimension. To achieve consistency among different brain sections from different mice, the brains were systematically sectioned as shown in Supplementary Figure 2A. This was accomplished by creating two vertical lines going through the most dorsomedial portion of the lateral ventricle and the most lateral portion of the corpus callosum. A horizontal line was also drawn at the most dorsomedial point of the lateral ventricle. A fourth line was drawn from the most ventral tip of the lateral ventricle to the most ventral visible point of the corpus callosum. The four lines plus the corpus callosum were the boundaries of the striatum for measurement purposes. A triangle at the intersection of two perpendicular lines above the ventricle was used to sample corpus callosum intensity. The cortex was sampled from a rectangle between two vertical lines representative of M1 layers V–VI. The striatum was sectioned into four quadrants by calculating the maximum length and width and drawing horizontal and vertical lines at the midpoint. The mean fluorescence integrated density and area were measured for each brain region and striatal quadrant using ImageJ software. Since the different ROIs have different areas, in order to compare fluorescence integrated density across the different regions, data was normalized based on the area of the region where fluorescence was measured, therefore integrated density data was divided by the area of each individual region and obtained data was referred to as fluorescence intensity.

#### Nearest neighbor distance (NND) and clustering

The Nearest Neighbor Distance (NND) plugin on ImageJ was used (v1.51j8; National Institute of Health, Bethesda, MD, United States) on epifluorescent images with no z-dimension. NND was calculated using methods described in González Ibanez et al. (2019) and used in multiple other publications (Tohyama et al., 1983; Keeley et al., 2020; Bordeleau et al., 2022; Zhang and Johnson, 2022). Using ImageJ, the Paint tool was used to place a 10 pixel width black dot in the center of each astrocyte. The center of each astrocyte was identified relative to its nucleus and equidistant from the brightest tips of their processes. The Thresholding tool was used to highlight only the black dots, effectively reducing each astrocyte to the dot at its center. Once the image was reduced to a white background with black dots, the analyze particle function on ImageJ was used to count and select all dots. Next, the NND plugin on ImageJ was used to calculate the distance between each dot and the nearest one (NNDs). The obtained NNDs were averaged to get the average NND per image. To define GFAP^+^ astrocyte clusters, we used maximum projections of confocal images acquired with a 16 μm z-dimension and condensed using the Z Project function on ImageJ at max intensity. We thresholded the GFAP channel to 70 and 255 with the dark background option checked. We then used the analyze particles function to put all GFAP^+^ areas into the ROI manager, and used the Or (Combine) function on the ROI manager to select all GFAP^+^ areas. Following this, we used the Enlarge option and enlarged the GFAP^+^ area by 10 pixels, which created boundaries around GFAP^+^ areas. We defined a GFAP^+^ cluster of astrocytes as a group of GFAP^+^ astrocytes containing more than one astrocyte that are grouped together within a neighboring distance no greater than 10 μm from the perimeters of each astrocyte (total distance 20 μm). Areas containing individual astrocytes that were not in close proximity to nearby astrocytes (within 20 μm) were removed from the ROI prior to performing the analyses. The analyze particle’s function tool was used in order to keep individual ROIs that could be removed if they corresponded to a single isolated astrocyte not in a cluster and no size criteria for remaining ROIs was applied.

#### HTT aggregation

For EM48 puncta analysis in 18-month-old mice, we used maximum projections of confocal images acquired with a 16 μm z-dimension and 1 μm step size, condensed using the Z Project function on Image J at max intensity, as previously described (Jeon et al., 2016; Sciacca and Cicchetti, 2017; Burrus et al., 2020; Wertz et al., 2020; Abjean et al., 2023; Gangwani et al., 2023). Images were converted to 8-bit using ImageJ and the threshold was set to 30 and 255 with the dark background option checked. The Analyze Particles function was used to count puncta. To measure HTT aggregation within and outside GFAP clusters we used the enlarged “cluster” area obtained from clustering analyses as a new ROI, measured its area, and opened it on the EM48 channel image. We then used the Clear function or the Clear Outside functions to remove puncta within or outside of GFAP^+^ astrocyte clusters, respectively. We then analyzed the number of EM48 puncta and divided by the cluster area or non-cluster area (area of entire image—cluster area). For analysis of puncta in WM, each fascicle was traced and added as an ROI. We then used the OR (Combine) function on the ROI manager to select all fascicles, and we added that to the ROI as well. After this step, the puncta analysis was analogous to that of the clusters—we used Clear or Clear outside and then measured EM48 puncta.

### Experimental design and statistical analyses

Data from three brain slices per animal were averaged together to get an animal average, and data per animal was used to conduct statistical analyses. Specific sample sizes are included in each figure legend. Data are expressed as Mean ± SEM, analyzed for statistical significance, and displayed by Prism 9 software (GraphPad, San Diego, CA, USA). To test normality, the D’Agostino and Pearson test was used when sample size was greater than or equal to 8 and the Shapiro-Wilk test was used when sample size was less than 8. Normal distributions were compared with *t*-test (two-tailed), ordinary one-way ANOVA with Tukey’s multiple comparisons test or two-way ANOVA with Sidak’s multiple comparison test when appropriate. The accepted level of significance was *p* ≤ 0.05, therefore samples indicated as n.s. (no significance) did not reach *p* ≤ 0.05.

## Results

### Reactive astrocyte subtypes exhibit differences in number and distribution throughout the striatum in zQ175 mice

**FIGURE 1:**
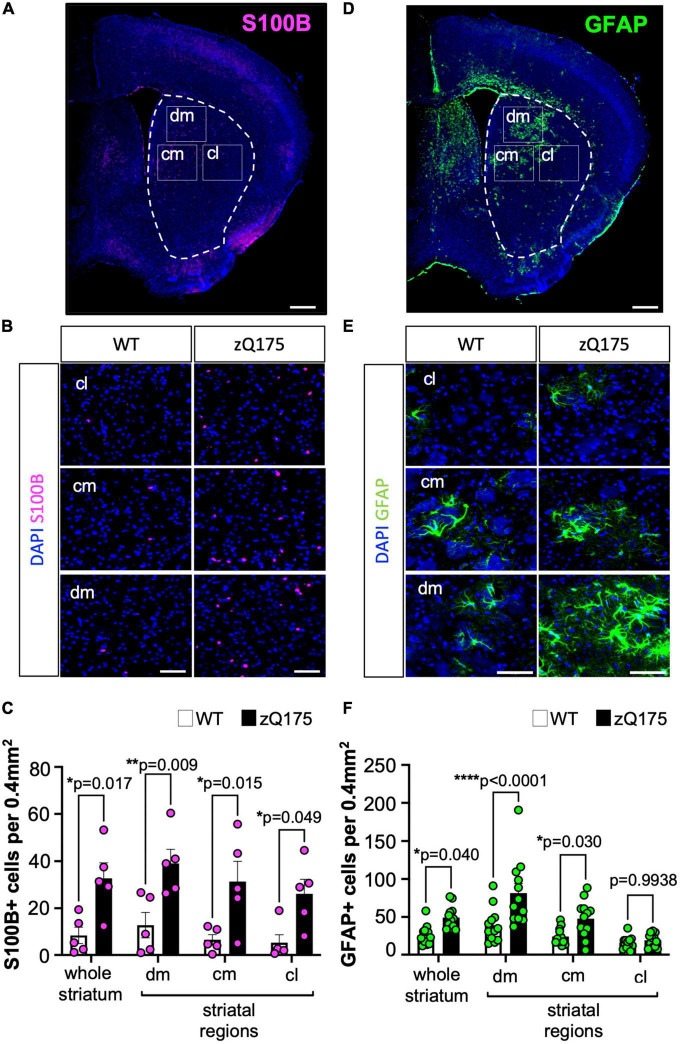
Number of GFAP^+^ and S100B^+^ astrocytes is differentially increased throughout the striatum of zQ175 mice. **(A)** Representative coronal section of the immunostaining of S100B and DAPI (stain nuclei) of the zQ175 mouse brain. The dotted line represents the striatum and rectangles represent dorsomedial (dm), centromedial (cm), and centrolateral (cl) regions. Scale bar, 500 μm. **(B)** Representative S100B immunostaining in dm, cm, and cl regions of the striatum in 12-month-old WT and zQ175 mice. DAPI stains nuclei. Scale bar, 50 μm. **(C)** S100B^+^ astrocytes number per 0.4 mm^2^ in whole striatum and in striatal regions (*n* = 5 mice/genotype) analyzed from images in **(B)**. **(D)** Representative coronal section of the immunostaining of GFAP and DAPI (stain nuclei) of the zQ175 mouse brain. Scale bar, 500 μm. **(E)** Representative GFAP immunostaining in dm, cm, and cl regions of the striatum in 12-month-old WT and zQ175 mice. Scale bar, 50 μm. **(F)** GFAP^+^ astrocyte number per 0.4 mm^2^ in whole striatum and in striatal regions (*n* = 12 mice/genotype). Number of cells in the whole striatum is calculated as the average of all three regions. Error bars denote mean ± SEM. Two-way ANOVA with Sidak’s multiple comparisons [**(D)** Interaction: *F* = 0.08170, df = 3; striatal region: *F* = 1.157, df = 3; genotype: *F* = 37.04, df = 1. **(F)** Interaction: *F* = 3.391, df = 3; striatal region: *F* = 16.10, df = 3; genotype: *F* = 28.04, df = 1]. Only *p*-values for comparisons between WT and zQ175 are shown. *p < 0.05, **p < 0.01, ****p < 0.0001.

Increased astrogliosis is a hallmark of HD. As increasing evidence for multiple subtypes of astrocytes with potential distinct functions arises (Khakh and Deneen, 2019; Al-Dalahmah et al., 2020; Hasel et al., 2021), the necessity for determining whether specific subtypes of astrocytes are differentially involved in HD becomes more essential. To investigate whether HD-related astrogliosis affects different astrocyte subtypes, we utilized two different IF markers of reactive astrocytes, S100B and GFAP, which each correspond to a different group of astrocytes as characterized by recent scRNA-seq studies (Al-Dalahmah et al., 2020). S100B is considered a marker of mature astrocytes and inflammation and expression of GFAP is associated with reactive astrocytes (Buffo et al., 2008; Kamphuis et al., 2012; Anlauf and Derouiche, 2013; Hol and Pekny, 2015; Escartin et al., 2021). We performed IF on coronal brain sections from WT and zQ175 mice at 12 months. At this age, zQ175 mice show robust motor deficits, and therefore this is considered a symptomatic time point (Heikkinen et al., 2012; Menalled et al., 2012; Yu et al., 2022). We assessed astrocyte number, expressed as the number of cells per 0.4 mm^2^, in three specific striatal locations (subregions) that correspond to dorsomedial (dm), centromedial (cm), and centrolateral (cl) for GFAP and S100B (Figure 1).

We found that there was an increased number of S100B^+^ astrocytes per imaged area in zQ175 mice compared to WT in both data from dm, cm, and cl that was averaged together (whole striatum), and when comparing within specific striatal regions (Figures 1A–C), demonstrating an increase in S100B^+^ astrocytes throughout the striatum in zQ175. This was consistent with previous reports indicating that neurodegeneration in HD occurs throughout the entire striatum (Bates et al., 2015; Morigaki and Goto, 2017). GFAP^+^ astrocytes also increased in number in the whole striatum in zQ175 when compared with WT (Figures 1D–F). However, when looking at GFAP^+^ astrocytes by different striatal areas in zQ175 mice compared to WT, the number of GFAP^+^ astrocytes was significantly increased only in the dm and cm striatum (Figures 1D–F), regions heavily affected in HD (Vonsattel et al., 1985), which accounted for the total increased number observed in the whole striatum analysis. When comparing striatal regions within each genotype, we observed a significant increase in GFAP^+^ cells only in zQ175 mice, in the dm vs. cm (*p* = 0.0013) or cl (*p* < 0.0001) and in the cm vs. the cl (*p* = 0.0085) (Figure 1F). Samples sizes for GFAP analyses allowed for additional sex-differences assessment (Supplementary Figure 1). We observed no significant differences between males and females when groups were compared within each genotype, WT (*n* = 5 males, 7 females) or HD mice (*n* = 6 males, 6 females). When comparing groups between WT and zQ175 we observed a trend toward increased number of GFAP^+^ astrocytes in zQ175 females (*p* = 0.0649) and a significant increase in zQ175 males (*p* = 0.0465) when compared with WT males and females, respectively, for the whole striatum (Supplementary Figure 1). No significant sex differences were found when data was analyzed per striatum region except for zQ175 females in the cm (*p* = 0.043). Overall, this data indicates that the overall difference in the number of GFAP^+^ astrocytes between WT and zQ175 cannot be explained by a sex effect. In addition, the increase in astrocyte number, in either S100B or GFAP, is unlikely to be related to neuronal loss or changes in the striatum volume, as previous studies in zQ175 mice revealed no differences in these parameters up to 22 months of age (Deng et al., 2021; Yu et al., 2022).

To further confirm the striatal regional differences in GFAP^+^ astrocytes, we utilized a macro zoom microscope to capture entire coronal slices and measured the fluorescence intensity for GFAP (Supplementary Figure 2). We used a systematic brain compartmentalization approach to define the area of different brain regions from where fluorescence intensity was measured, and we sampled from the corpus callosum (CC) and motor cortex M1 layers V-VI (Ctx) (Supplementary Figure 2A). We also subdivided the striatum into four quadrants corresponding, relatively, to cm, cl, dm, and dl (dorsolateral). GFAP mean fluorescence intensity was unchanged in the CC and Ctx, in zQ175 mice compared to WT (Supplementary Figures 2B, C). Within the striatum, GFAP fluorescence intensity was specifically increased in dm striatum in zQ175 mice compared to WT, while no significant changes were observed in dl, cm, or cl striatum (Supplementary Figures 2B, C). These results confirmed a unique spatial distribution of GFAP^+^ astrocytes preferentially occurring in the dm striatum of HD mice and suggested a differential response of different reactive astrocyte subtypes in both number and distribution throughout the striatum in zQ175 mice.

**FIGURE 2:**
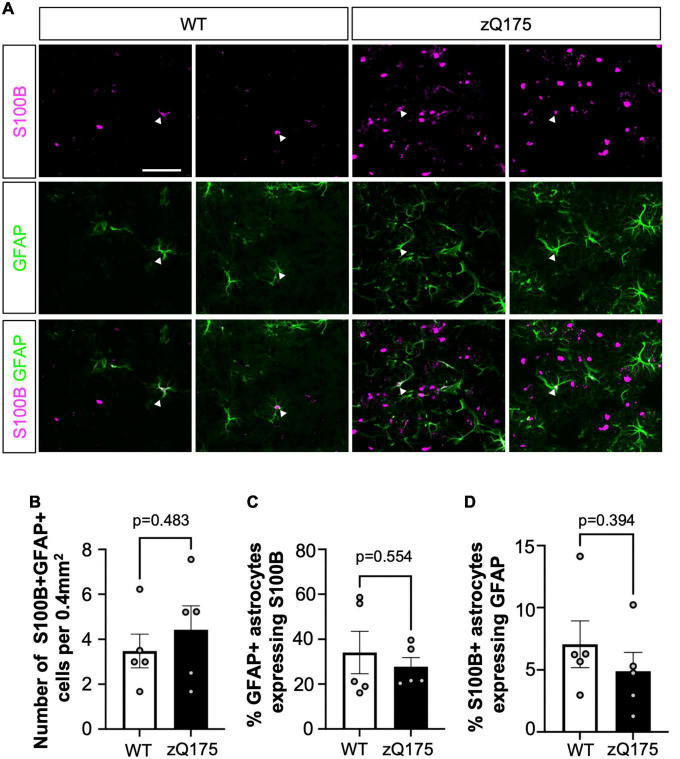
Dual GFAP^+^S100B^+^ astrocytes constitute a small portion of striatal astrocytes, and their number is not altered in zQ175 mice. **(A)** Representative images from 12-month-old WT and zQ175 mice showing S100B and GFAP immunofluorescence in the dorsal striatum. White arrows indicate colocalization. **(B)** Number of S100B^+^ GFAP^+^ astrocytes per 0.4 mm^2^ in the striatum of WT and zQ175 mice at 12 months (*n* = 5 mice/genotype). **(C)** Percent GFAP^+^ astrocytes that express S100B. **(D)** Percent S100B^+^ astrocytes that express GFAP. Scale bar, 50 μm. Error bars denote mean ± SEM. Un-paired Student’s *t*-test [**(B)** *t* = 0.7343, df = 8; **(C)** *t* = 0.6163, df = 8; **(D)** *t* = 0.8994, df = 8].

In order to determine if S100B^+^ and GFAP^+^ astrocytes corresponded to distinct subpopulations, we assessed the colocalization between the two astrocyte markers (Figure 2). Surprisingly, we found very little colocalization. The number of co-stained GFAP^+^S100B^+^ astrocytes was less than ∼5 astrocytes per 0.4 mm^2^ area (<10% of total GFAP^+^ and S100B^+^) throughout the striatum in both WT and zQ175 mice, with no significant differences between genotypes (Figures 2A–D). When assessing the percentage of GFAP^+^ astrocytes throughout the striatum we found ∼35% expressed S100B (Figure 2C) and only ∼6% of S100B^+^ astrocytes expressed GFAP (Figure 2D). This data, in combination with our findings indicate that GFAP^+^ and S100B^+^ astrocytes preferentially accumulate in different regions of the striatum and supports the idea that these are two distinct populations of astrocytes differentially affected in HD striatum with a unique spatial compartmentalization for GFAP^+^ astrocytes.

### GFAP^+^ astrocytes are clustered in the dorsomedial striatum of zQ175 mice and are associated with low mHTT aggregate load

Previous analyses suggested that astrocyte somata are evenly distributed throughout any given brain region and that their processes overlap only minimally, indicating that each astrocyte covers an exclusive territory of neuropil and maintain a constant distance with their corresponding neighbors (Bushong et al., 2002; Grosche et al., 2013). However, reactive astrocytes have previously been shown to group around protein aggregates and sites of injury presenting a non-uniform distribution (Buffo et al., 2008; Kamphuis et al., 2012). Spatially grouped astrocytes are typically associated with focal inflammation and/or neurodegeneration. They can coordinate with microglia to remove dying neurons, with astrocytes specifically engulfing the diffuse apoptotic bodies derived from distal dendritic branches of dying neurons (Damisah et al., 2020). With the increased regional-specificity and heterogeneous distribution of GFAP^+^ vs. S100B^+^ astrocytes within the striatum of zQ175 mice, we studied whether GFAP^+^ and S100B^+^ astrocytes presented a clustering pattern. Although the term “cluster” (spatially speaking) for astrocytes has not been previously used, we used this term inspired by previous reports in which cellular spatial clustering of other types of cells has been described (Jiao et al., 2022; Pagès et al., 2022).

**FIGURE 3:**
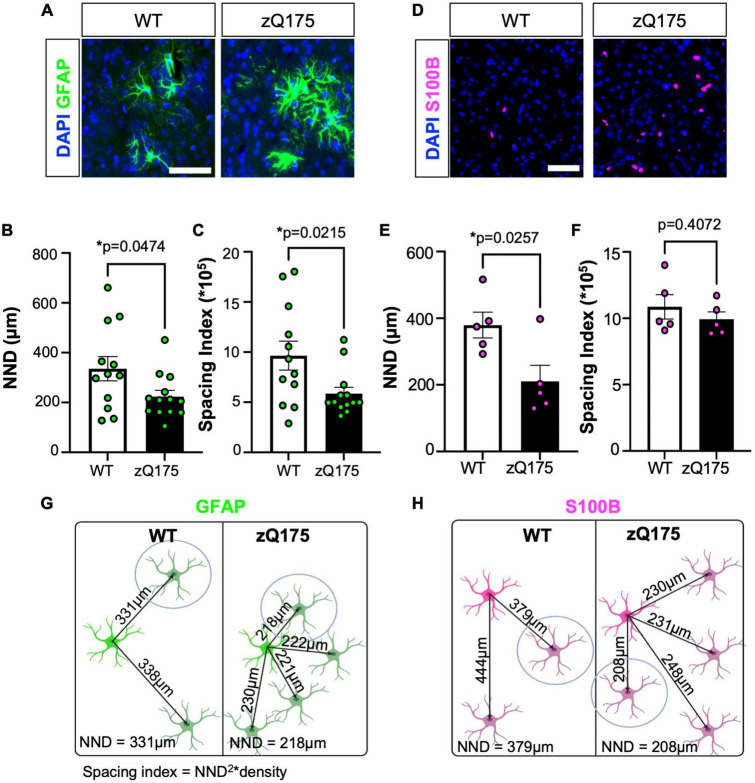
GFAP^+^ astrocytes in the dm striatum of zQ175 mice show increased clustering. **(A)** Representative immunostaining for GFAP in the striatum of WT and zQ175 mice at 12 months. DAPI stains nuclei. Scale bar, 50 μm. NND **(B)** and spacing index (NND^2^ × density) **(C)** between GFAP^+^ astrocytes was calculated (*n* = 12–13 mice/genotype). **(D)** Representative immunostaining for S100B in the striatum of WT and zQ175 mice at 12 months. DAPI stains nuclei. Scale bar, 50 μm. NND **(E)** and spacing index (NND^2^ × density) **(F)** between S100B^+^ astrocytes was calculated (*n* = 5 mice/genotype). Diagram representing changes in clustering for GFAP **(G)** and S100B **(H)** astrocytes. The cell circled in blue is the nearest neighbor. Created on BioRender.com. Error bars denote mean ± SEM. Un-paired Student’s *t*-test [**(B)** *t* = 2.772, df = 23; **(C)** *t* = 2.425, df = 23; **(E)** *t* = 2.733, df = 8; **(F)** *t* = 0.8748, df = 8] *p < 0.05.

To determine astrocyte clustering, we first started calculating the nearest neighbor distance (NND), which measures the shortest distance from the center of one cell to the center of its closest neighboring cell (Tohyama et al., 1983; González Ibanez et al., 2019; Keeley et al., 2020; Bordeleau et al., 2022; Zhang and Johnson, 2022; Supplementary Figures 3A–C). We found that the mean NND for GFAP^+^ astrocytes in zQ175 mice was significantly lower (∼30%) than in WT mice (Figures 3A, B). To discard those changes in NND were due to increased number of astrocytes seen in zQ175 mice (Figures 1D–F), we calculated the spacing index (NND^2^ × density) from mean NND values to account for changes in the total number of cells per any given area. The spacing index, together with the NND are parameters to determine cell proximity. The smaller the NND and spacing index are, the closer the cells are, which we interpreted as a higher likelihood to grouping into clusters. The spacing index was significantly lower in zQ175 mice compared to WT (Figure 3C), further confirming that GFAP^+^ astrocytes were clustered in zQ175 mice. No sex differences in the NND or spacing index for GFAP^+^ astrocytes were observed (Supplementary Figures 3D, E). Taken together, not only we observed increased number and specialized striatal compartmentalization of GFAP^+^ astrocytes but also increased clustering, suggesting the presence of specific foci within the striatum that induce astrocyte reactivity. We performed similar analyses for S100B^+^ astrocytes and while we observed decreased NND in zQ175 mice compared to WT (Figures 3D, E), the spacing index was not significantly different (Figure 3F). This indicates that the decreased NND of S100B^+^ astrocytes was merely due to increased number, as seen in Figures 1A, B, and that these astrocytes do not cluster. This provides further evidence that clustering is a specific characteristic of GFAP^+^ astrocytes in zQ175 mice (Figures 3G, H).

**FIGURE 4:**
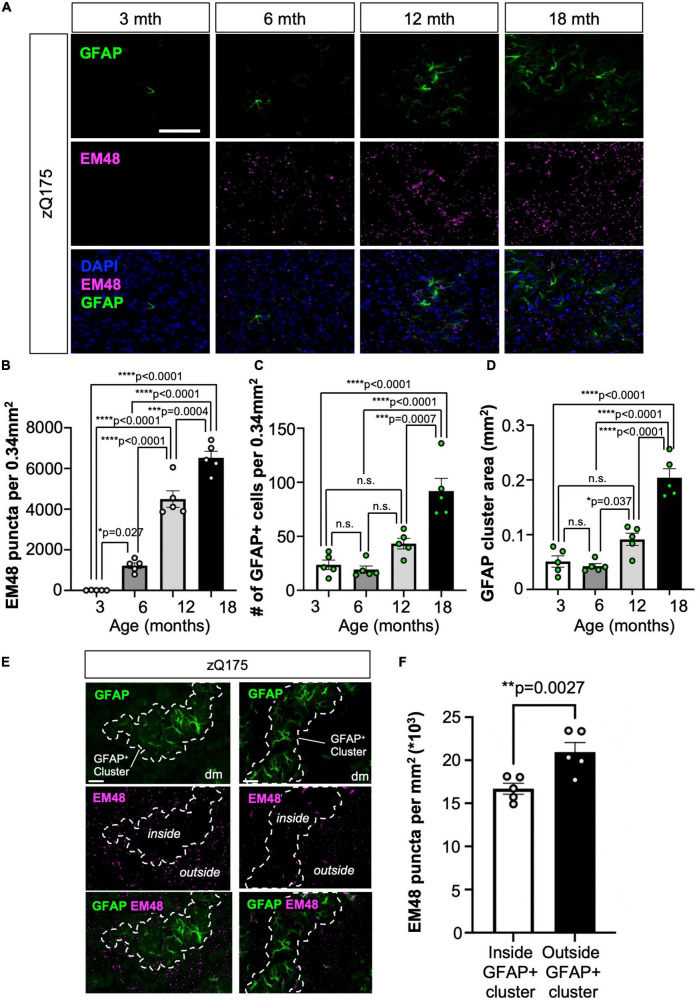
GFAP^+^ astrocytes clustered in dm striatum with low HTT aggregate load in zQ175 mice. **(A)** Representative images from zQ175 mice at 3, 6, 12, 18, and 22 months showing GFAP and HTT aggregation (EM48) in the dm striatum. DAPI stains nuclei. Scale bar is 100 μm. Number of EM48 puncta per 0.34 mm^2^ **(B)**, number of GFAP^+^ cells per 0.34 mm^2^ **(C)**, and the mean area (mm^2^) of GFAP^+^ astrocyte clusters **(D)** in the striatum of 3-, 6-, 12-, and 18-month-old zQ175 mice analyzed using confocal images (*n* = 5 mice/age). **(E)** Representative immunofluorescence images showing GFAP and EM48 in the dm striatum of zQ175 mice at 18 months. Dotted lines surround GFAP^+^ astrocyte clusters occupying areas of low HTT aggregate load. Scale bar is 50 μm. **(F)** Number of EM48 puncta per μm^2^ inside GFAP^+^ clusters and outside GFAP^+^ clusters in 18 month old zQ175 mice analyzed using confocal images (*n* = 5 mice/genotype). Ordinary one-way ANOVAs with Tukey’s multiple comparisons test [**(B)** *F* = 123.4, df = 3; **(C)** *t* = 22.91, df = 3; **(D)** *t* = 42.27, df = 3] and Paired student’s *t*-test [**(F)** *t* = 6.591, df = 4]. Error bars denote mean ± SEM. *p < 0.05, **p < 0.01, ***p < 0.001, ****p < 0.0001. *p*-values > *p* = 0.05 are shown as n.s.

We then hypothesized that GFAP^+^ clusters in HD could be associated with areas of increased HTT aggregation and neurodegeneration. We defined a GFAP^+^ cluster of astrocytes as a group of GFAP^+^ astrocytes containing more than one astrocyte that are grouped together within a neighboring distance no greater than 10 μm from the perimeters of each astrocyte. By defining a 10 μm distance criteria from the perimeter of each astrocyte (total distance 20 μm) we ensured that if two astrocytes were within that distance or less, there would not be physical space to be occupied by another astrocyte and they were grouped as part of a cluster (Supplementary Figure 4A). We first confirmed that enhanced GFAP^+^ astrocyte number and clustering within the dm striatum coincided with the appearance and progressive accumulation of HTT aggregates. We used ImageJ to produce perimeters around groups of GFAP^+^ astrocytes that were within a 10 μm distance from another astrocyte (Supplementary Figures 4B, C). We performed longitudinal IF analyses for GFAP and EM48 (marker for HTT aggregates) (Gutekunst et al., 1999) in the striatum of zQ175 mice at 3, 6, 12, and 18 months old (Figures 4A–D). We observed that HTT aggregates started to accumulate at 6 months, as previously described (Yu et al., 2022), and progressively increased at older ages (Figure 4B). GFAP^+^ astrocyte number as well as cluster size also increased with age (Figures 4C, D). We also compared GFAP^+^ astrocyte number and cluster size between dm and cl at different ages and found a significant increase in GFAP^+^ astrocyte number at 12 and 18 months and cluster size at 3, 12, and 18 months in the dm vs. cl (Supplementary Figures 5A, B). In addition, we found that starting at 12 months there were significantly less aggregates in areas covered by GFAP^+^ astrocyte clusters than areas outside of clusters, and that difference continued progressively up to 18 months (age *F* = 234.1, cluster *F* = 12.33) (Figures 4E, F and Supplementary Figures 5C–E). A recent report showed that reactive astrocytes foster mHTT clearance in neurons and improve neuronal defects by promoting proteostasis (Abjean et al., 2023). Therefore, it is possible that GFAP^+^ astrocyte clusters in HD participate in decreasing HTT aggregate load in certain regions of the striatum in HD.

### GFAP^+^ astrocytes are associated with white matter fascicles traveling through the dm striatum

**FIGURE 5:**
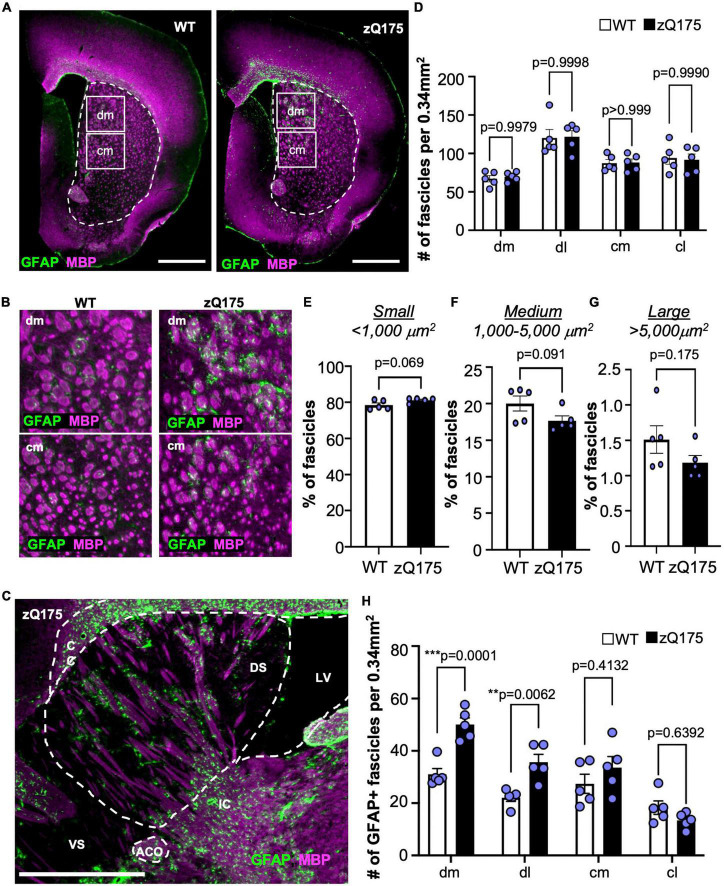
GFAP^+^ astrocytes are associated with WM fascicles in the dm striatum. **(A)** GFAP and MBP immunofluorescence in coronal brain sections from 18-month-old WT and zQ175 mice. The striatum is shown with a dotted line. Scale bar is 1 mm. **(B)** Zoomed images from the dm (upper panel) and cm (lower panel) regions of the striatum from images in **(A)**. **(C)** Immunofluorescence of a sagittal brain section from an 18-month-old zQ175 mouse costained with GFAP and MBP. Corpus callosum (CC), dorsal striatum (DS), ventral striatum (VS), lateral ventricle (LV), and anterior commissure (ACO). Scale bar is 1 mm. **(D)** Number of fascicles per 0.34 mm^2^ in striatal regions of 18-month-old WT and zQ175 mice. Percent of WM fascicles in the striatum with a cross-sectional area lower than 1,000 μm^2^ [small–**(E)**], 1,000–5,000 μm^2^ [medium–**(F)**], or greater than 5,000 μm^2^ [large–**(G)**] in 18-month-old WT and zQ175 mice. **(H)** Number of GFAP^+^ fascicles per 0.34 mm^2^ in striatal regions 18-month-old WT and zQ175 mice. Whole striatum values were calculated as the average of all four striatum regions. All experiments were carried out with *n* = 5 mice/genotype and all analysis was done using confocal images. Two-way ANOVA with Sidak’s multiple comparisons [**(D)** Interaction: *F* = 0.04568, df = 3; striatal region: *F* = 20.06, df = 3; genotype: *F* = 0.01802, df = 1. **(H)** Interaction: *F* = 6.900, df = 3; striatal region: *F* = 26.67, df = 3; genotype: *F* = 18.61, df = 1] and un-paired student’s *t*-tests [**(E)** *t* = 2.098, df = 8; **(F)** *t* = 1.922, df = 8; **(G)** *t* = 1.488, df = 8]. For two-way ANOVAs, only *p*-values for comparisons between WT and zQ175 are shown. Error bars denote mean ± SEM. **p < 0.01, ***p < 0.001.

We explored if GFAP^+^ clusters within the dorsal striatum were associated with specific anatomical structures. Considering the large size of GFAP^+^ clusters (Figure 4D) and the reduced EM48 immunostaining within those clusters in the dorsal striatum (Figure 4F), we reasoned that the areas occupied by GFAP^+^ clusters could correspond to white matter (WM) fascicles. WM changes have previously been reported in patients with HD and are relevant pathophysiological features (de la Monte et al., 1988; Rosas et al., 2006, 2010), although reports in mouse models of HD are lacking. Therefore, we performed IF analyses for GFAP and myelin basic protein (MBP), a marker of WM in coronal (Figures 5A, B) and sagittal (Figure 5C) slices from WT and zQ175 mice at 18 months old. We first assessed whether there were changes in the amount of WM present in each striatal subregion (Figures 5A–D). We found the greatest area occupied by WM was in the dm striatum (∼30%) and the lowest ventrally in the cl (∼15%) for both WT and zQ175 mice (Figures 5A–C and Supplementary Figures 6A–D). No significant differences between genotypes were observed except for the dl striatum where zQ175 mice showed lower percent area occupied by WM than WT mice (Supplementary Figure 6). We also analyzed the total number of WM fascicles throughout the striatum and within striatal regions (Figure 5D and Supplementary Figures 6F, G). The dl striatum showed the greatest number of fascicles and the dm striatum the lowest number of fascicles in both genotypes (WT dl vs. WT dm *p* < 0.0001, WT dl vs. WT cm *p* = 0.0115, zQ175 dl vs. zQ175 dm *p* < 0.0001, zQ175 dl vs. zQ175 cm *p* = 0.0092) (Figure 5D and Supplementary Figures 6F, G) but no significant differences were found between WT and zQ175 mice (Figure 5D). We then analyzed the average cross-sectional area of WM fascicles throughout the striatum and found no significant differences between genotypes (Supplementary Figure 6). After classifying all WM fascicles into small (<1,000 μm^2^), medium (1,000–5,000 μm^2^) and large (>5,000 μm^2^) we found a trend toward increased percentage of small fascicles in zQ175 compared to WT (*p* = 0.069) and trend toward decreased% in medium size fascicles (*p* = 0.091) (Figures 5E–G). Altogether, these data suggested a subtle decrease in the size and in the area of the striatum covered by WT fascicles in zQ175 mice which could be indicative of modest WM alterations in this mouse model.

**FIGURE 6:**
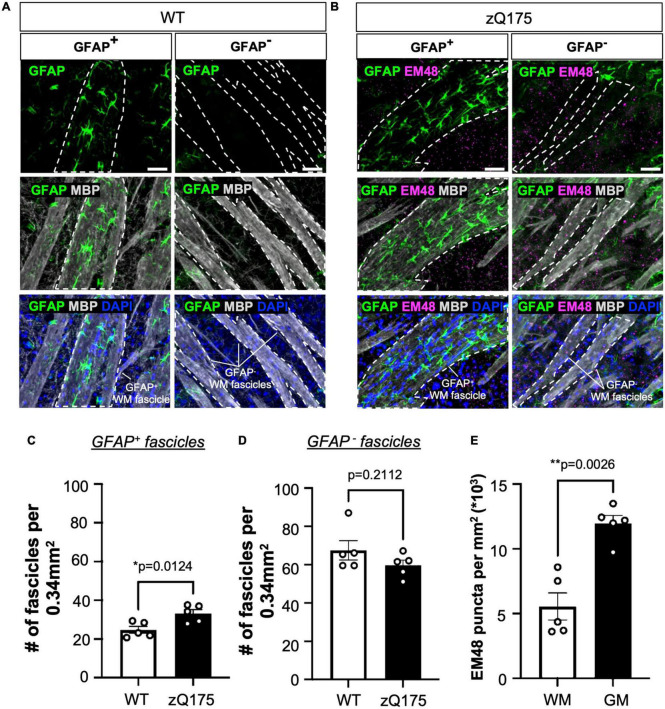
White matter (WM) fascicles are associated with areas of low aggregate load. GFAP and EM48 immunofluorescence on sagittal sections from 18-month-old WT **(A)** and zQ175 **(B)** mice. Scale bar is 50 μm. Number of GFAP^+^ **(C)** and GFAP^–^ **(D)** fascicles per 0.34 mm^2^ in whole striatum of 18-month-old WT and zQ175 mice (*n* = 5 mice/genotype). **(E)** Number of EM48 puncta per mm^2^ inside WM fascicles and in gray matter (GM) in 18-month-old zQ175 mice (*n* = 5 mice/genotype). All analysis was done using confocal images. Paired [**(E)** *t* = 6.701, df = 4] and un-paired [**(C)** *t* = 3.212, df = 8; **(D)** *t* = 1.359, df = 8] student’s *t*-tests. Error bars denote mean ± SEM. *p < 0.05, **p < 0.01.

When assessing the colocalization between GFAP and MBP, we found a significant increase in the number of GFAP^+^ WM fascicles in the dm and dl striatum of zQ175 compared to WT (Figure 5H and Supplementary Figures 6H, I). When comparing across striatal regions within each genotype, the dm striatum had significantly more GFAP^+^ fascicles than the dl (*p* = 0.0054), cm (*p* = 0.0012), and cl (*p* < 0.0001) only in zQ175 mice (Figure 5H). A sagittal view showed GFAP^+^ astrocytes within the dm were positioned along the WM fascicles (Figure 5C). Through increased magnification, we confirmed that in both WT and zQ175 mice, there were GFAP^+^ and GFAP^–^ WM fascicles in the striatum (Figures 6A, B and Supplementary Figures 7A, B). Throughout the whole striatum, there were more GFAP^+^ fascicles in zQ175 mice compared to WT while no significant changes were seen in GFAP^–^ fascicles between genotypes (Figures 6C, D). Interestingly, compared to striatal gray matter (GM), we found that all WM fascicles, both GFAP^+^ and GFAP^–^, corresponded to areas of low HTT aggregate load in zQ175 mice compared to gray matter area of the striatum (Figures 6B, E).

**FIGURE 7:**
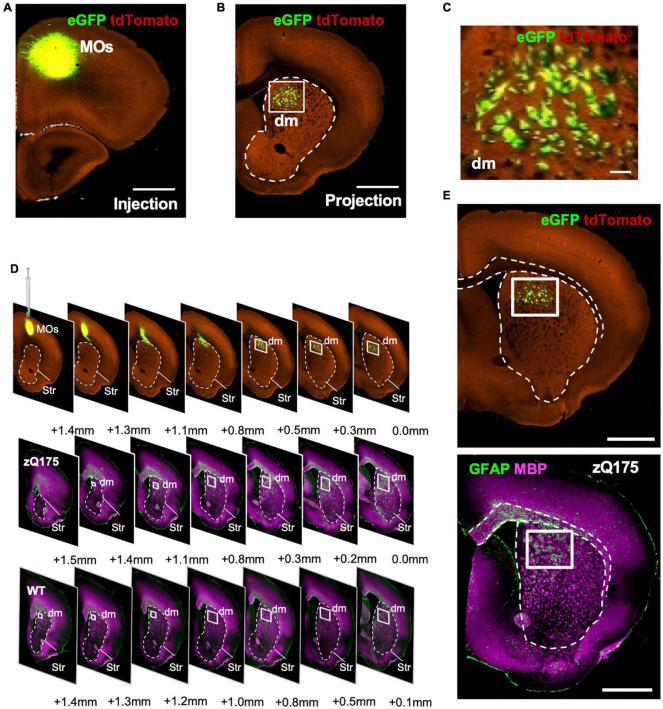
Motor area (MOs) WM fascicles project into the dm striatum and accumulate in areas with enhanced GFAP^+^ clusters. eGFP tracing in Syt6-Cre-TdTomato mice showing MOs injection location **(A)** and projections through the dm striatum **(B)** from Allen Mouse Brain Connectivity Atlas: connectivity.brain-map.org/projection/experiment/177459319. **(C)** Zoomed images from the dm regions of the striatum boxed in **(B)**. Comparison of serial coronal sections **(D)** or brain bregma + 3.5 mm **(E)** for MOs-eGFP injected Syt6-Cre-TdTomato mice, and 18-month-old zQ175 immunostained with GFAP and MBP. WT mice are used as reference. Striatum is highlighted with a dotted line and dm striatum is denoted with a white box. Serial coronal sections for MOs-eGFP injected Syt6-Cre mice in G were obtained from the Allen Mouse Brain Connectivity Atlas: connectivity.brain-map.org/projection/experiment/122642490. Scale bar is 1 mm.

Striatal fascicles contain fibers projecting between the cerebral cortex and the thalamus and brainstem; they are the homolog of the better-encapsulated primate internal capsule bundle. Because these fascicles are known to have some topographic organization (Coizet et al., 2017), we wondered if this spatial selectivity could reflect fascicles traveling through the dm striatum from specific cortical brain areas. Using the Mouse Connectivity feature of the Allen Brain Atlas (Allen Mouse Brain Connectivity Atlas), we performed a source search with the striatum as the target, as well as a search through all of the injections in the database from relevant cortical areas. We determined that the most likely source of these fascicles is the secondary motor area (MOs) (Figures 7A–C). Tract-tracing experiments (Experiments 277957202, 177459319, and 122642490) reveal that MOs fascicles labeled with eGFP traverse the dm striatum (Figures 7A–C and Supplementary Figures 8A–F). In contrast, the primary motor area (MOp) projects through more lateral regions and is not as concentrated in the dm striatum as the projections from MOs (Allen Mouse Brain Connectivity Atlas, Experiment 277957908) (Supplementary Figure 8H). We also investigated projections from all other frontal areas (the frontal pole, anterior cingulate area, prelimbic area, infralimbic area, and orbital areas) and found that they project to more medial and ventral fascicles within the striatum (Supplementary Figures 8I–P). A side-by-side comparison using serial coronal immunostaining for GFAP-MBP from WT, zQ175 mice and anterograde tracing from mice injected in MOs clearly show that WM fascicles projecting through the dm striatum presented the same pattern as to that observed for GFAP^+^ clusters (Figures 7D, E). Therefore, we conclude that accumulation of mHTT in the striatum of HD mice induces a spatial distribution of GFAP^+^ astrocytes within the dm striatum most likely associated with WM fascicles projecting from MOs to the striatum, thalamus, and brainstem.

## Discussion

Numerous studies have addressed the importance of astrocytes in HD (Liévens et al., 2001; Behrens et al., 2002; Faideau et al., 2010; Lee et al., 2013; Khakh et al., 2017; Diaz-Castro et al., 2019; Wood et al., 2019; Al-Dalahmah et al., 2020; Yu et al., 2022). However, our understanding of the differential susceptibility of different astrocyte subtypes in HD is still limited. We conducted a simultaneous comprehensive analysis of the abundance and distribution of two subtypes of astrocytes (S100B^+^ and GFAP^+^) in symptomatic zQ175 mice. We demonstrated that different astrocytes subtypes respond differently during disease conditions in terms of astrogliosis, spatial distribution, and clustering. Our data not only provided crucial information for understanding astrocyte pathology in HD but also highlighted the importance of proper astrocyte marker selection and accurate definition of striatum regions of interest when studying astrocyte biology in HD.

We have used two astrocyte markers in our study, S100B and GFAP. These markers were chosen based on their association with reactive astrocytes (Mrak and Griffin, 2001; Villarreal et al., 2014; Liddelow et al., 2017; Hagmeyer et al., 2019; Michetti et al., 2019; Escartin et al., 2021) and based on a previous scRNA-seq study in which each marker was specific to one transcriptomic group of astrocytes in HD (Al-Dalahmah et al., 2020). These subpopulations of astrocytes are not entirely distinct, but rather probably belong on a spectrum of expression, as they can exhibit partial colocalization (Mack et al., 2018; Klein et al., 2020; Du et al., 2021), which was recapitulated in our study. While we acknowledge that partial overlapping between these two different markers exist, less than 10% of the total evaluated astrocytes showed colocalization between the two astrocyte markers and the number of those GFAP^+^S100B^+^ did not differ between WT and HD mice. Our study focused on evaluating those astrocytes that were either GFAP^+^ or S100B^+^ and validated that they represent two overall distinct populations differentially altered in HD.

S100B is involved in several functions that range between cell survival, protein phosphorylation, cytoskeletal dynamics, and intracellular Ca^2+^ homeostasis (Michetti et al., 2019). S100B is increased in several neurodegenerative diseases, where it has been postulated to be induced by microglial cytokines and to cause both intracellular and extracellular effects that lead to neurotoxicity (Sheng et al., 1994; Mrak and Griffin, 2001; Sathe et al., 2012; Serrano et al., 2017). We found S100B^+^ astrocytes to be homogeneously distributed throughout the striatum with an increased number in zQ175 mice compared to WT. The homogeneity of S100B^+^ related astrogliosis is consistent with the widely accepted idea that striatal degeneration occurs throughout the striatum and is also consistent with increased striatal inflammation, as previously reported (Serrano et al., 2017).

Glial fibrillary acidic protein marks intermediate filaments in astrocytes, and it is important for motility and structural stability of the cell (Eng et al., 2000; Hol and Pekny, 2015). Many studies point to GFAP^+^ astrocytes as a reactive subtype that is particularly enhanced in response to injury or inflammation (Buffo et al., 2008; Kamphuis et al., 2012; Hol and Pekny, 2015; Escartin et al., 2021). GFAP levels are also enhanced in fibrous astrocytes associated with WM compared to protoplasmic astrocytes associated with gray matter (Eng et al., 1971), although it is unclear the implications of such differential expression. Recent studies support the functional enrichment of GFAP and S100B genes in HD, which are seen in different HD mouse models, R6/2 and zQ175, and in human-derived cells (Benraiss et al., 2021). However, past HD studies are inconsistent in their use of GFAP as a marker for striatal astrogliosis due to this marker’s “patchy” pattern in the striatum. Our data demonstrated that GFAP “patchy” immunostaining is indeed characteristic of GFAP^+^ striatal astrocyte pathology and that it is consistently enhanced in the dm striatum. A potential explanation for such localized accumulation of GFAP^+^ astrocytes could be its association with neurodegeneration and/or behavioral alterations. MSN loss is widely reported in postmortem brains of patients with HD (Vonsattel et al., 1985), although no significant changes in the number of MSNs have been observed in zQ175 or other mouse models of HD (Sun et al., 2002; Naver et al., 2003; Yu et al., 2022). Therefore, accumulation of GFAP^+^ astrocytes within the dm striatum may not be directly related to cell death. The dm domain of the striatum is understood to be partially responsible for instantiating goal-directed behaviors (Hunnicutt et al., 2016; Lipton et al., 2019; Balleine et al., 2021). Interestingly, a reduction in goal-directed behaviors, which is associated with apathy, is a common psychiatric symptom in HD (Thompson et al., 2002). Therefore, it is possible that accumulation of GFAP^+^ astrocyte clusters within the dm striatum of HD mice contributes to this behavioral alteration.

We originally hypothesized that GFAP^+^ astrocyte clustering may indicate specific areas that enhance astrocyte recruitment, such as areas of focal inflammation, synaptic degeneration, or HTT aggregation. Contrary to what we expected, we found that there were fewer mHTT aggregates in areas of GFAP^+^ clusters. Interestingly, GFAP^+^ astrocyte clusters were found surrounding or within WM fascicles traveling through the dm striatum. Previous studies in patients with HD have demonstrated the dysregulation of WM in the striatum (de la Monte et al., 1988; Gutekunst et al., 1999; Rosas et al., 2006; Bates et al., 2015; Casella et al., 2020; Gabery et al., 2021) and our data showed modest alterations in the size of WM fascicles and in the percent area of the striatum covered by WM which could be indicative of subtle WM dysregulation. Postmortem analyses of HD patient brains have also shown low aggregate load in WM tracts (Gutekunst et al., 1999; Herrmann et al., 2021), which is consistent with our findings. In whole brain analyses, WM astrocytes have stronger GFAP expression than gray matter astrocytes (Lundgaard et al., 2014; Werkman et al., 2020) and while that may explain the GFAP^+^ astrocytes found within the WM fascicles, it does not account for the specific accumulation of GFAP^+^ astrocytes surrounding WM fascicles in the dm striatum. One possibility is that association of GFAP^+^ clusters of astrocytes with WM fascicles seals them off from the rest of the striatum in order to protect them from mHTT-mediated damage. It is evident from our data that only certain fascicles show GFAP^+^ astrocyte association. Based on comparisons between our GFAP-MBP data and data from the Atlas Brain mapping frontal WM fascicles in the mouse brain, we identified that MOs fibers were projecting through the dm striatum in the region where a higher number of GFAP^+^ astrocytes was found. We therefore speculated GFAP^+^ clusters of astrocytes are associated with MOs WM fascicles. MOs axons terminate broadly and topographically throughout the striatum (Reep and Corwin, 1999; Hintiryan et al., 2016). MOs fibers present in the striatum, however, are not bound exclusively for the striatum, but also constitute the homolog of the primate internal capsule, with fibers projecting to the thalamus and brainstem. These appear to be concentrated more medially, as might be expected given the location of the MOs. Rodent MOs is likely homologous to primate premotor areas, such as pre-supplementary motor area (pre-SMA), SMA, and/or MOs (Estrada-Sánchez and Rebec, 2013; Gremel and Costa, 2013), and these regions are also involved in goal-directed behaviors (Gremel and Costa, 2013). Premotor areas have been shown to be involved in HD, as functional magnetic resonance imaging of pre-symptomatic and early symptomatic HD patients performing repeated finger movements showed dysfunction of these brain areas (Klöppel et al., 2009; Minkova et al., 2015). Furthermore, studies in postmortem samples from late stages of HD also show significant cell loss in these regions (Macdonald and Halliday, 2002; Thu et al., 2010). Studies in mouse models have revealed the importance of protecting MOs cortico-striatal function by showing that MOs cortex-dorsolateral striatum stimulation reverses motor symptoms and synaptic deficits in the R6/1 mouse model (Fernández-García et al., 2020). While the causal role for MOs cortico-striatal dysfunction in HD is unclear, it is possible that pathological accumulation of GFAP^+^ astrocytes around those WM fascicles contributes to such dysregulation.

In summary, our study provided new insights on the differential alteration, both at the level of number and spatial distribution, of two distinct populations of astrocytes in the striatum of the zQ175 mouse model. Our data also highlights a potential connection between the pathological accumulation of GFAP^+^ astrocytes within the dm striatum and MOs WM fascicles. Our analyses implicate MOs fibers, but there may be other regions involved, particularly thalamic and brainstem regions that project to MOs (among others). Therefore, studies that combine tract-tracing with GFAP labeling in HD and WT mice will be necessary to confirm specific connections involved with GFAP^+^ clustering and the dm striatum. Other future studies involving stereological analyses for accurate counting of cell densities, alternative animal models, 3D spatiotemporal analyses and astrocyte reporter mouse lines such as Bac aldh1l1-eGFP or eaat2-tdT (Yang et al., 2011; Winchenbach et al., 2016) will be crucial to confirm our findings. Finally, our study did not evaluate the functional implications of GFAP^+^ astrocyte clustering and therefore specific manipulations of distinct astrocytes subpopulations are needed for a full understanding of the dynamics of astrocyte heterogeneity and their contribution to HD.

## Data availability statement

The original contributions presented in this study are included in the article/Supplementary material, further inquiries can be directed to the corresponding author.

## Ethics statement

This animal study was reviewed and approved by the University of Minnesota Institutional Animal Care and Use Committee (IACUC) in compliance with the National Institutes of Health guidelines for the care and use of laboratory animals under the approved animal protocol 2007-A38316.

## Author contributions

RG-P obtained funding for the study. RG-P and TB designed the experiments. NZ harvested mice used in the study. TB, MT, and JV performed IF and imaging. TB and MT analyzed the data. SH and DH performed anatomical comparisons. TB wrote the first draft of the manuscript. All authors edited subsequent versions, read, and approved the final version of the manuscript.

## Supplementary material

Supplementary Figure 1 Increased GFAP^+^ astrocyte number in zQ175 mice is not influenced by sex differences. GFAP^+^ astrocyte number per 0.4 mm^2^ in male and female WT and zQ175 mice in the whole striatum **(A)**, dm **(B)**, cm **(C)**, and cl **(D)** (*n* = 5–7/sex/genotype). Two-way ANOVA with Sidak’s multiple comparisons [**(A)** Interaction: *F* = 0.06543, df = 1; genotype: *F* = 11.26, df = 1; sex: *F* = 0.7841, df = 1. **(B)** Interaction: *F* = 0.7669, df = 1; genotype: *F* = 4.568, df = 1; sex: *F* = 1.527, df = 1. **(C)** Interaction: *F* = 0.04847, df = 1; genotype: *F* = 9.885, df = 1; sex: *F* = 0.04207, df = 1. **(D)** Interaction: *F* = 1.680, df = 1; genotype: *F* = 0.5444, df = 1; sex: *F* = 0.2786, df = 1]. Error bars denote mean ± SEM. **p* < 0.05, ***p* < 0.01, ****p* < 0.001, *****p* < 0.0001.

Supplementary Figure 2 GFAP^+^ astrocyte fluorescence intensity is preferentially increased in the cortex and dm striatum of zQ175 mice. **(A)** Systematic brain compartmentalization approach used for fluorescence intensity analyses in the motor cortex M1 layer V-VI (Ctx), corpus callosum (CC), and striatal quadrants (dl, dm, cm, cl). The striatum is outlined with a dotted white line. DAPI stains nuclei. **(B)** Representative image of GFAP fluorescence at 1.6X zoom in 12-month-old WT and zQ175 mice. Scale bar is 1 mm. **(C)** GFAP fluorescence intensity in WT and zQ175 brains (*n* = 5 mice/genotype). Fluorescence intensity was calculated using FIJI. Data was relativized to 100% WT. Two-way ANOVA with Sidak’s multiple comparisons [**(C)** Interaction: *F* = 0.7074, df = 5; striatal region: *F* = 0.7070, df = 5; genotype: *F* = 11.55, df = 1]. Only *p*-values for comparisons between WT and zQ175 are shown. Error bars denote mean ± SEM. **p* < 0.05, ***p* < 0.01, ****p* < 0.001, *****p* < 0.0001.

Supplementary Figure 3 Increased GFAP^+^ astrocyte clustering in zQ175 mice is not influenced by sex differences. **(A)** Representative GFAP^+^ astrocytes showing dot placement for NND calculation. Scale bar is 10 μm. **(B,C)** Representative GFAP^+^ astrocytes showing actual distance between cells. Scale bar is 10 μm. GFAP^+^ astrocyte NND **(D)** and spacing index **(E)** in male and female WT and zQ175 mice in the whole striatum (*n* = 5–7/sex/genotype). Two-way ANOVA with Sidak’s multiple comparisons [**(D)** Interaction: *F* = 0.1467, df = 1; genotype: *F* = 3.762, df = 1; sex: *F* = 0.0.01328, df = 1. **(E)** Interaction: *F* = 1.165, df = 1; genotype: *F* = 6.942, df = 1; sex: *F* = 1.143, df = 1]. Error bars denote mean ± SEM. **p* < 0.05, ***p* < 0.01, ****p* < 0.001, *****p* < 0.0001.

Supplementary Figure 4 GFAP^+^ astrocyte clustering procedure and EM48 puncta analysis. **(A)** Representative GFAP^+^ astrocytes with 3 astrocytes clustering together and 1 isolated astrocyte. Red lines are 10 μm from the center of the astrocyte. Gray lines are the perimeter traces as defined through thresholding. The blue lines are the expanded perimeter after a 10 μm expansion. Scale bar is 10 μm. **(B)** Procedure for setting astrocyte perimeters to define GFAP^+^ astrocyte clustering. **(C)** Procedure for EM48 puncta analysis relative to GFAP^+^ astrocyte clusters. Screenshots from ImageJ are shown. Red arrowheads indicate isolated astrocytes that were removed from analysis.

Supplementary Figure 5 GFAP^+^ astrocyte clusters in zQ175 mice increase with age in the dm striatum and present low HTT aggregate load in zQ175 mice. Number of GFAP^+^ astrocytes per 0.34 mm^2^ **(A)** and average cluster area (μm^2^) **(B)** in the dm vs. cl striatum at 3, 6, 12, and 18 months in zQ175 mice (*n* = 5 mice/age). The number EM48 puncta per μm^2^ outside GFAP^+^ clusters **(C)**, inside GFAP^+^ clusters **(D)**, and compared together **(E)** in 3, 6, 12, and 18 month old zQ175 mice (*n* = 5 mice/age). Two-way ANOVA with Sidak’s multiple comparisons [**(A)** Interaction: *F* = 12.69, df = 3; age: *F* = 21.54, df = 3; striatal region: *F* = 71.01, df = 1. **(B)** Interaction: *F* = 26.20, df = 3; age: *F* = 41.59, df = 3; striatal region: *F* = 153.3, df = 1. **(E)** Interaction: *F* = 3.158, df = 3; age: *F* = 234.1, df = 3; striatal region: *F* = 15.33, df = 1] and ordinary one-way ANOVA with Tukey’s multiple comparisons test [**(C)** *F* = 123.2, df = 3; **(D)** *t* = 112.0, df = 3]. For two-way ANOVAs, only *p*-values for comparisons between WT and zQ175 are shown. All analysis was done using confocal images. Error bars denote mean ± SEM. **p* < 0.05, ***p* < 0.01, ****p* < 0.001, *****p* < 0.0001.

Supplementary Figure 6 Characterization of WM fascicles in WT and zQ175 mice. Percent of striatum occupied by WM in the dm **(A)**, dl **(C)**, cm **(B)**, and cl **(D)** striatum in 18 month WT and zQ175 mice (*n* = 5 mice/genotype). **(E)** Average cross-sectional area (μm^2^) of WM fascicles throughout the striatum in 18-month WT and zQ175 mice (*n* = 5 mice/genotype). Un-paired student’s *t*-tests [**(A)** *t* = 0.4401, df = 8; **(B)** *t* = 1.311, df = 8; **(C)** *t* = 2.430, df = 8; **(D)** *t* = 1.156, df = 8; **(E)** *t* = 1.825, df = 8]. All analysis was done using confocal images. Number of WM fascicles per 0.34 mm^2^ in striatal regions of WT **(F)** and zQ175 **(G)** mice. Number of GFAP^+^ WM fascicles per 0.34 mm^2^ in striatal regions of WT **(H)** and zQ175 **(I)** mice (*n* = 5 mice/age/genotype). Ordinary one-way ANOVA with Tukey’s multiple comparisons test [**(F)** *F* = 8.502, df = 3; **(G)** *t* = 12.41, df = 3; **(H)** *F* = 4.782, df = 3; **(I)** *t* = 25.96, df = 3]. All analysis was done using confocal images. Error bars denote mean ± SEM. **p* < 0.05, ***p* < 0.01, ****p* < 0.001, *****p* < 0.0001.

Supplementary Figure 7 GFAP^+^ astrocyte association with WM fascicles. GFAP and EM48 immunofluorescence on coronal sections from 18-month-old WT **(A)** and zQ175 **(B)** mice. DAPI stains nuclei. Left panels show GFAP^+^ and right panels show GFAP^–^ fascicles. Scale bars are 50 μm.

Supplementary Figure 8 Candidate fascicle origin locations and their projections through the striatum. **(A)** Diagram showing eGFP injection into MOs of Htr2-Cre mice. Created with BioRender.com. eGFP tracing showing MOs injection location in Htr2a-Cre mice **(B)** and projections through the striatum **(C)**. Allen Mouse Brain Connectivity Atlas: connectivity.brain-map.org/projection/experiment/277957202. **(D)** Diagram showing eGFP injection into MOs of Syt6-Cre mice. Created with BioRender.com. eGFP tracing showing MOs injection location in Syt6-Cre mice **(E)** and projections through the striatum **(F)**. Allen Mouse Brain Connectivity Atlas: connectivity.brain-map.org/projection/experiment/122642490. **(G)** Diagram showing eGFP injection into primary motor cortex (MOp) of Htr2-Cre mice. Created with Biorender.com. **(H)** eGFP tracing showing MOp injection location in Htr2-Cre mice and projections through the striatum. Allen Mouse Brain Connectivity Atlas: connectivity.brain-map.org/projection/experiment/277957908. **(I)** Diagram showing eGFP injection into anterior cingulate cortex (ACC) of Syt6-Cre mice. Created with BioRender.com. **(J)** eGFP tracing showing ACC injection location in Syt6-Cre mice and projections through the striatum. Allen Mouse Brain Connectivity Atlas: connectivity.brain-map.org/projection/experiment/125801033. **(K)** Diagram showing eGFP injection into infralimbic cortex (IL) of C57BL/6J. Created with BioRender.com. eGFP tracing showing IL injection location **(L)** in C57BL/6J mice and projections through the striatum **(M)**. Allen Mouse Brain Connectivity Atlas: connectivity.brain-map.org/projection/experiment/157556400. **(N)** Diagram showing eGFP injection into the prelimbic area (PrL) of Rbp4-Cre-TdTomato mice. Created with BioRender.com. eGFP tracing in Rbp4-Cre-TdTomato showing PrL injection location **(O)** and projections through the striatum **(P)** from Allen Mouse Brain Connectivity Atlas: connectivity.brain-map.org/projection/experiment/263106036. Scale bars are 1 mm.
