# Supplementary material for: Striatal spatial heterogeneity, clustering, and white matter association of GFAP+ astrocytes in a mouse model of Huntington’s disease
Source: Front Cell Neurosci. 2023 Apr 28;17:1094503. doi: 10.3389/fncel.2023.1094503 (PMC10175581; doi:10.3389/fncel.2023.1094503)
Supplement: Supplementary Figure 1 — Increased GFAP+ astrocyte number in zQ175 mice is not influenced by sex differences. GFAP+ astrocyte number per 0.4 mm2 in male and female WT and zQ175 mice in the whole striatum (A), dm (B), cm (C), and cl (D) (n = 5–7/sex/genotype). Two-way ANOVA with Sidak’s multiple comparisons [(A) Interaction: F = 0.06543, df = 1; genotype: F = 11.26, df = 1; sex: F = 0.7841, df = 1. (B) Interaction: F = 0.7669, df = 1; genotype: F = 4.568, df = 1; sex: F = 1.527, df = 1. (C) Interaction: F = 0.04847, df = 1; genotype: F = 9.885, df = 1; sex: F = 0.04207, df = 1. (D) Interaction: F = 1.680, df = 1; genotype: F = 0.5444, df = 1; sex: F = 0.2786, df = 1]. Error bars denote mean ± SEM. *p < 0.05, **p < 0.01, ***p < 0.001, ****p < 0.0001. [file Data_Sheet_1.PDF]

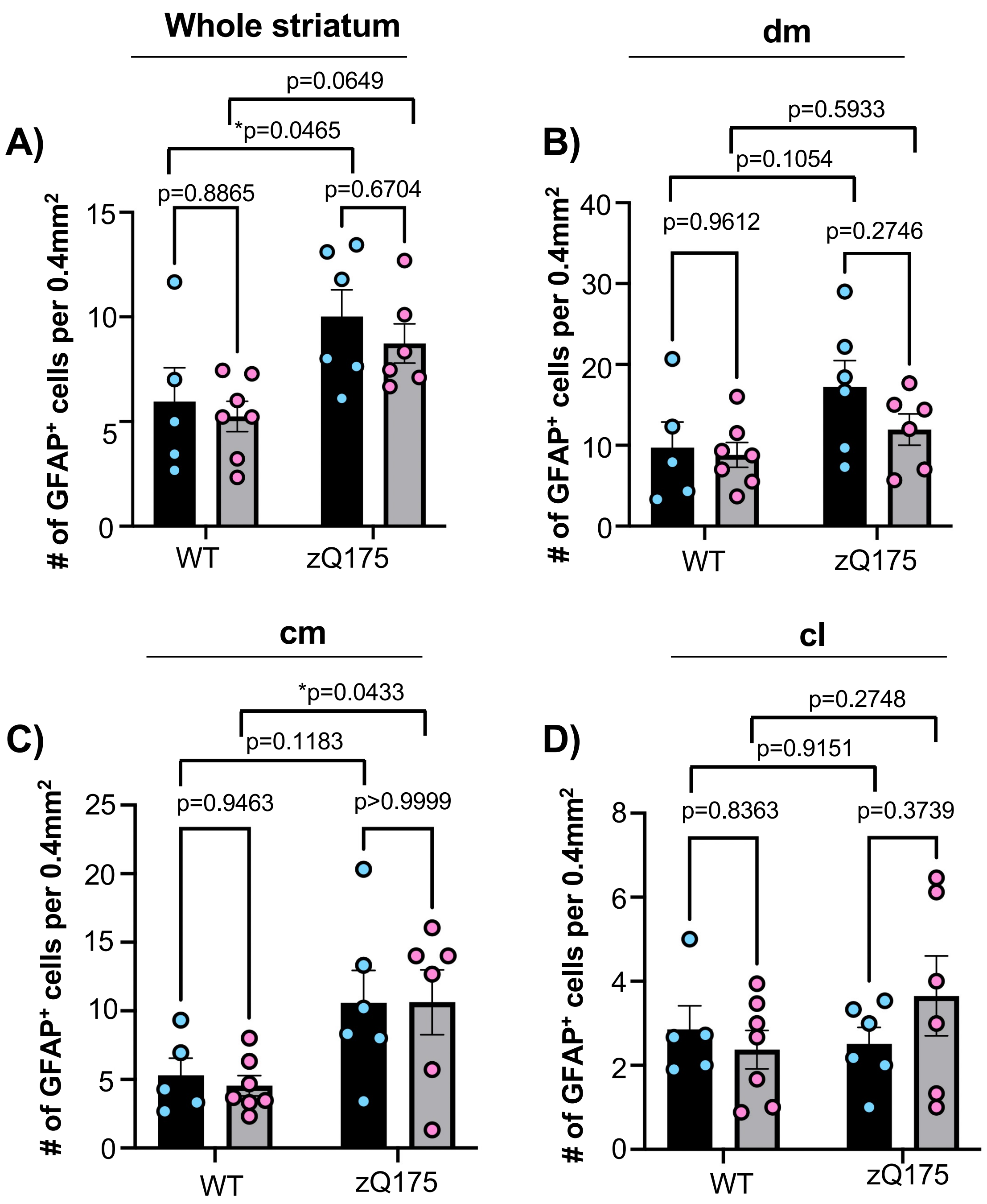
Whole striatum
A)
p=0.0649
*p=0.0465
p=0.8865
p=0.6704
# of GFAP+ cells per 0.4mm2
WT
zQ175
dm
B)
p=0.5933
p=0.1054
p=0.9612
p=0.2746
cm
C)
*p=0.0433
p=0.1183
p=0.9463
p>0.9999
cl
D)
p=0.2748
p=0.9151
p=0.8363
p=0.3739

Figure S1

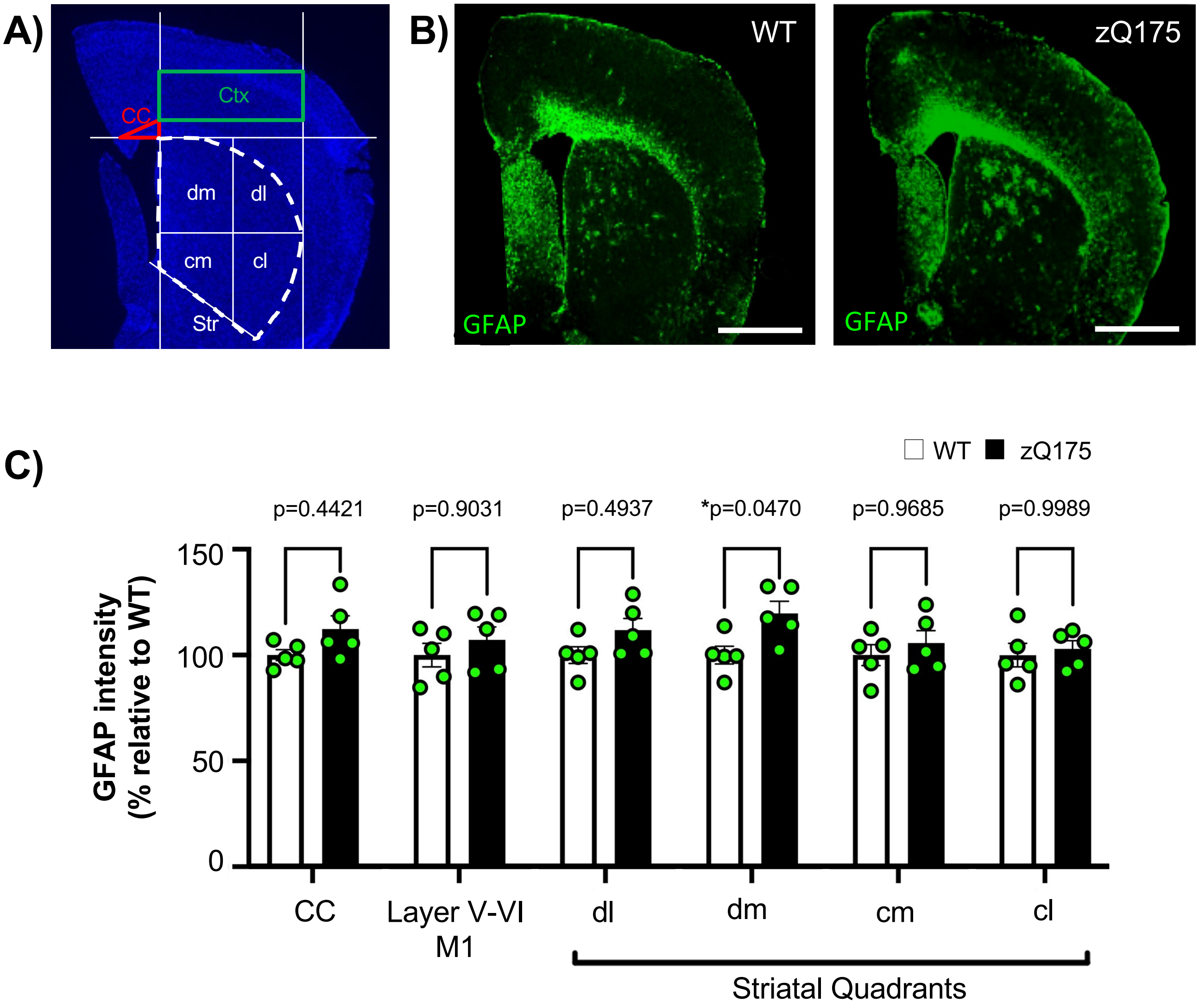
A)
Ctx
CC
dm
dl
cm
cl
Str
B)
WT
zQ175
GFAP
GFAP
C)
WT
zQ175
p=0.4421
p=0.9031
p=0.4937
*p=0.0470
p=0.9685
p=0.9989
GFAP intensity
(% relative to WT)
150
100
50
0
CC
Layer V-VI
M1
dl
dm
cm
cl
Striatal Quadrants

Figure S2

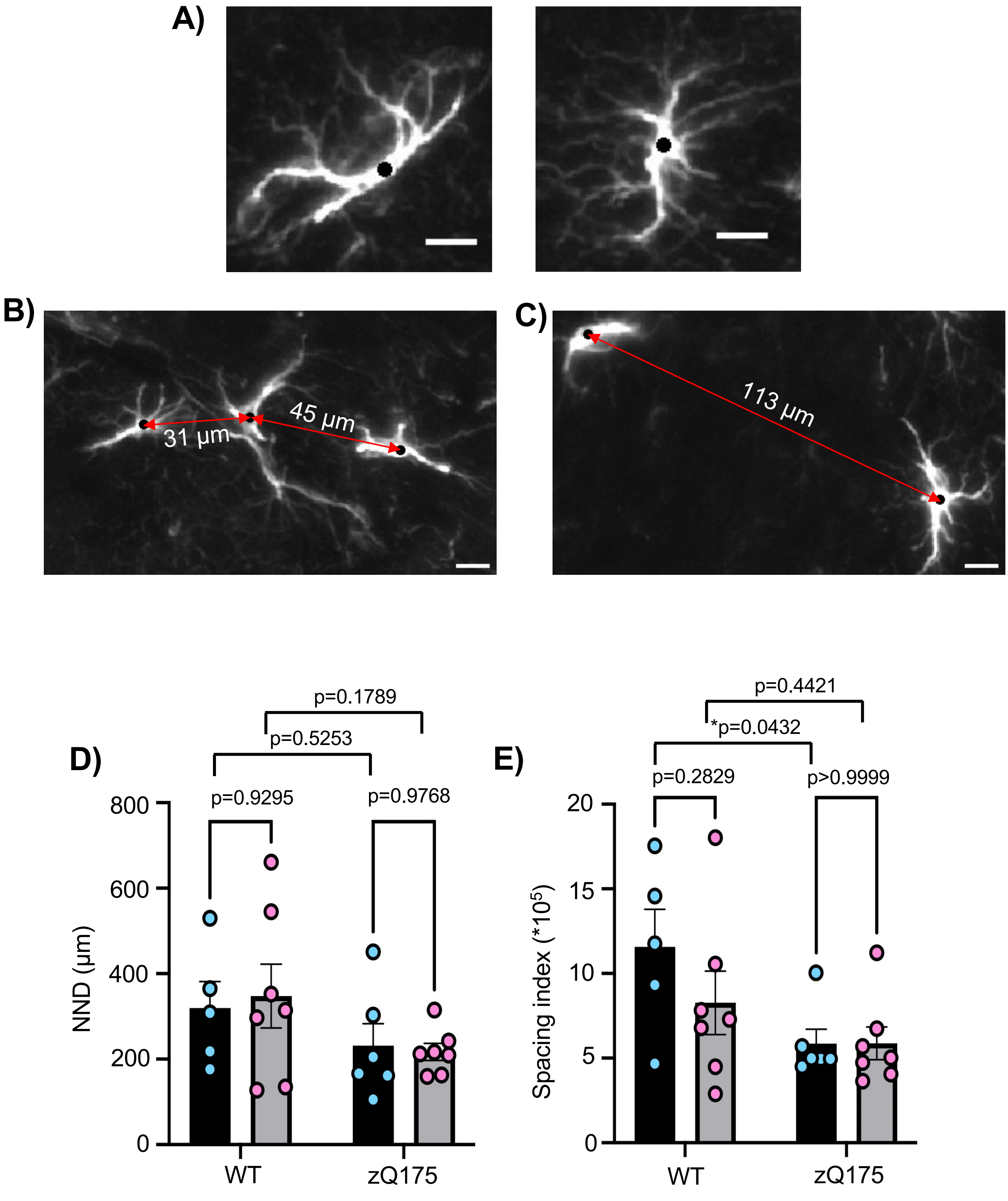
A)
B)
31 μm
45 μm
C)
113 μm
D)
p=0.1789
p=0.5253
p=0.9295
p=0.9768
800
600
400
200
0
NND (μm)
WT
zQ175
E)
p=0.4421
*p=0.0432
p=0.2829
p>0.9999
20
15
10
5
0
Spacing index (*10⁵)
WT
zQ175

Figure S3

A)

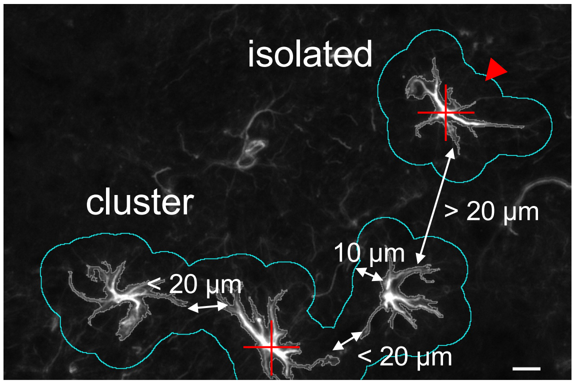

B) Setting astrocyte perimeters to define cluster areas

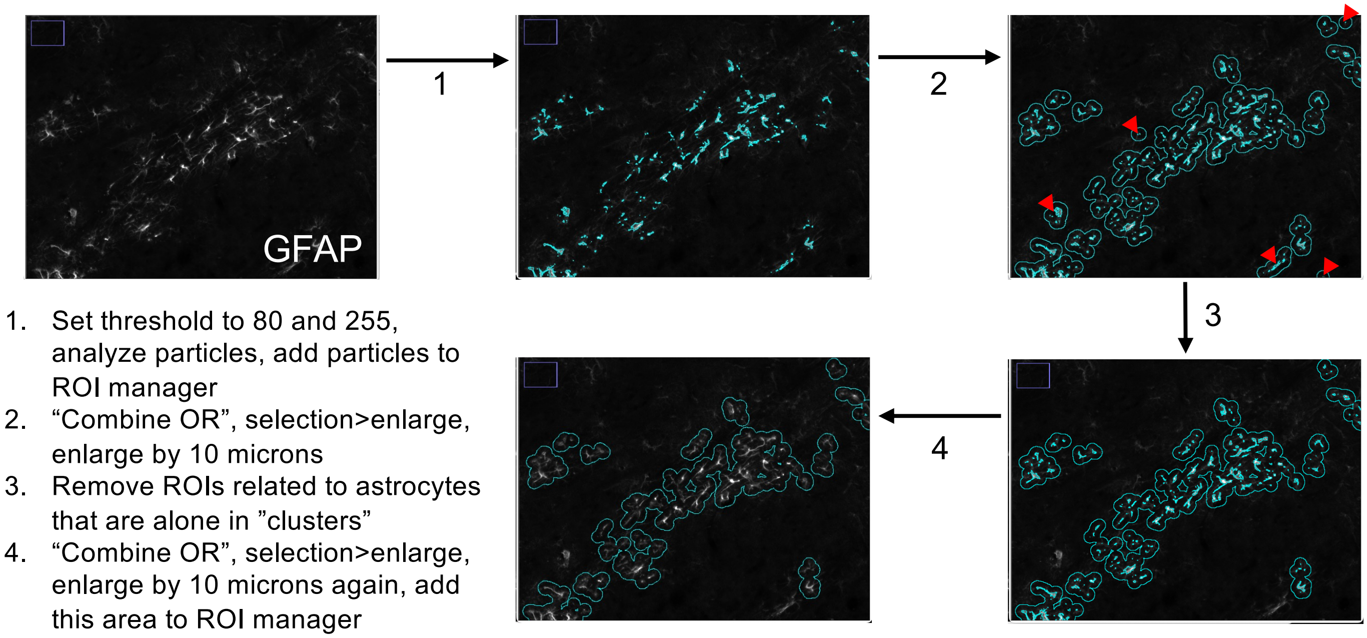

C) EM48 puncta analysis in astrocyte clusters

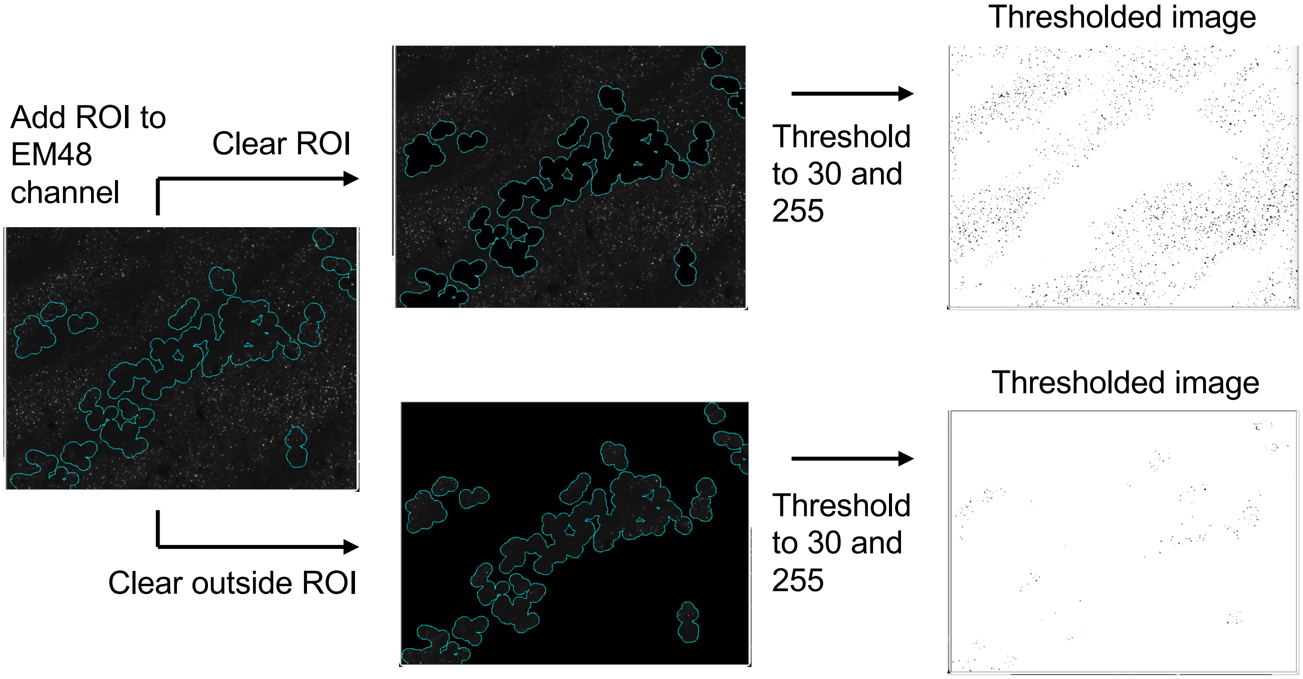

Figure S4

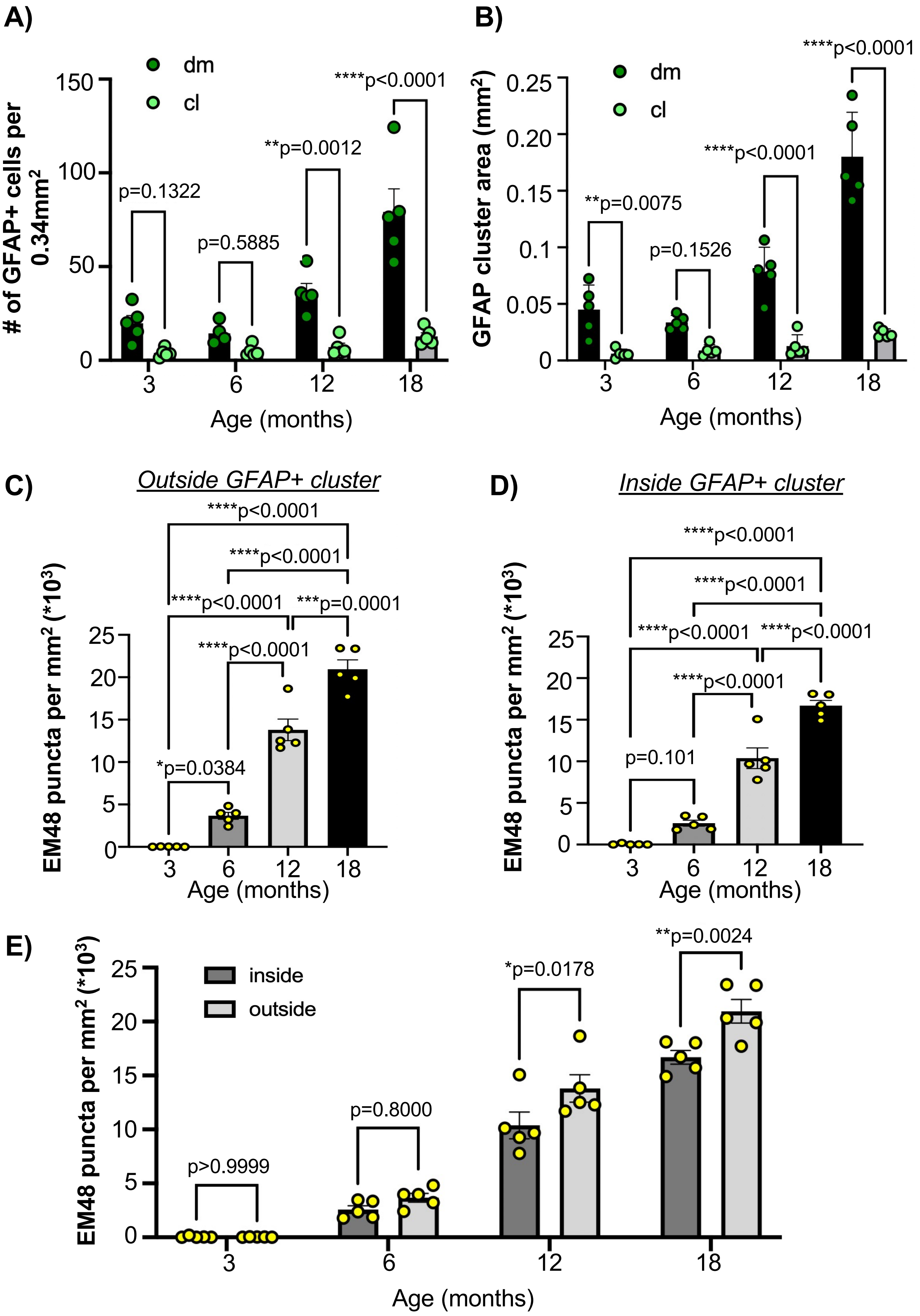

A)
dm
cl
# of GFAP+ cells per 0.34mm²
Age (months)
p=0.1322
p=0.5885
**p=0.0012
****p<0.0001
B)
GFAP cluster area (mm²)
**p=0.0075
p=0.1526
****p<0.0001
****p<0.0001
C)
Outside GFAP+ cluster
EM48 puncta per mm² (*10³)
*p=0.0384
****p<0.0001
***p=0.0001
D)
Inside GFAP+ cluster
p=0.101
****p<0.0001
E)
inside
outside
p>0.9999
p=0.8000
*p=0.0178
**p=0.0024

Figure S5

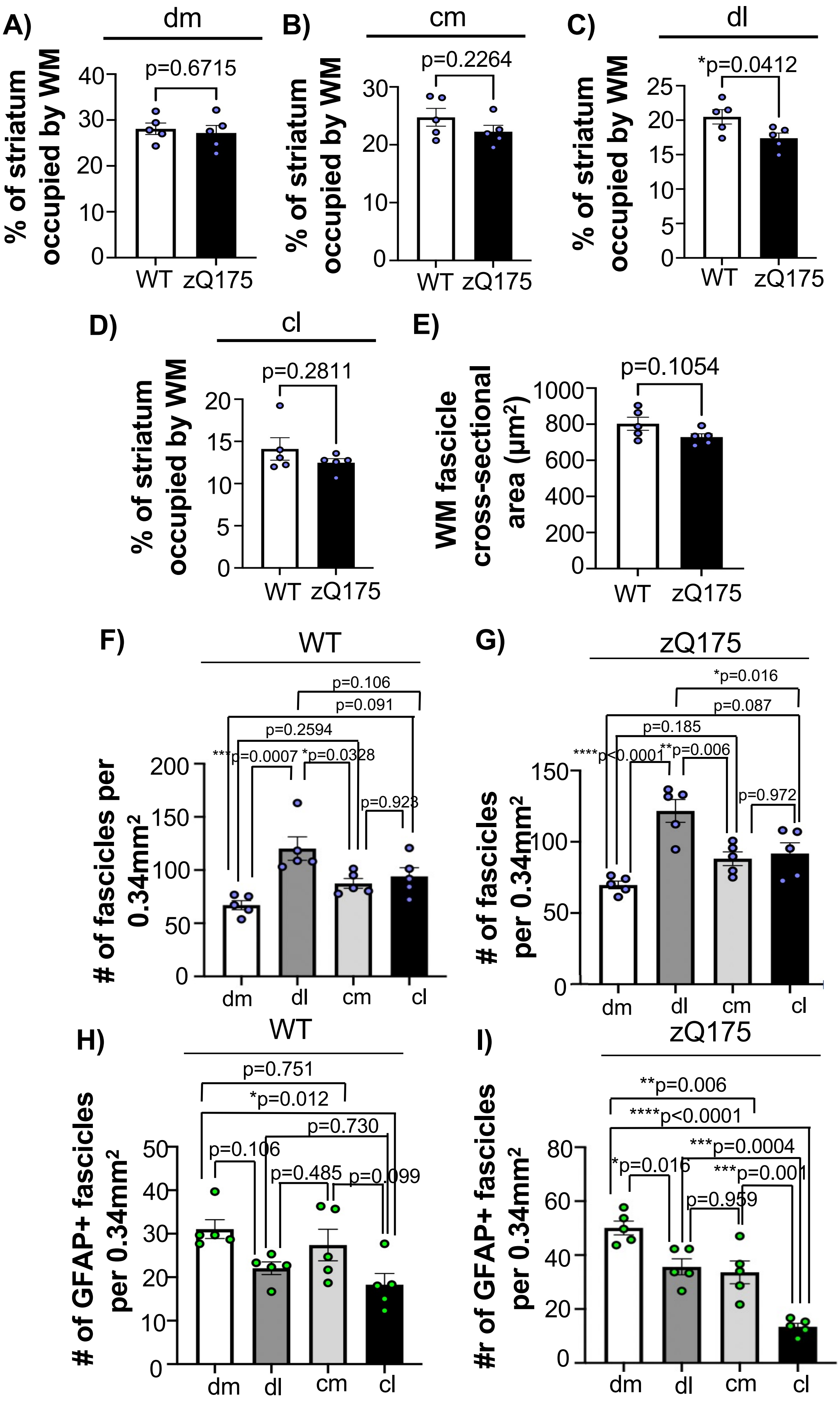
A)
dm
p=0.6715
% of striatum occupied by WM
WT
zQ175
B)
cm
p=0.2264
C)
dl
*p=0.0412
D)
cl
p=0.2811
E)
p=0.1054
WM fascicle cross-sectional area (µm²)
F)
WT
# of fascicles per 0.34mm²
p=0.106
p=0.091
p=0.2594
***p=0.0007
*p=0.0328
p=0.928
dm
dl
cm
cl
G)
zQ175
*p=0.016
p=0.087
p=0.185
****p<0.0001
**p=0.006
p=0.972
H)
WT
# of GFAP+ fascicles per 0.34mm²
p=0.751
*p=0.012
p=0.730
p=0.106
p=0.485
p=0.099
I)
zQ175
#r of GFAP+ fascicles per 0.34mm²
**p=0.006
****p<0.0001
***p=0.0004
*p=0.016
***p=0.001
p=0.959

Figure S6

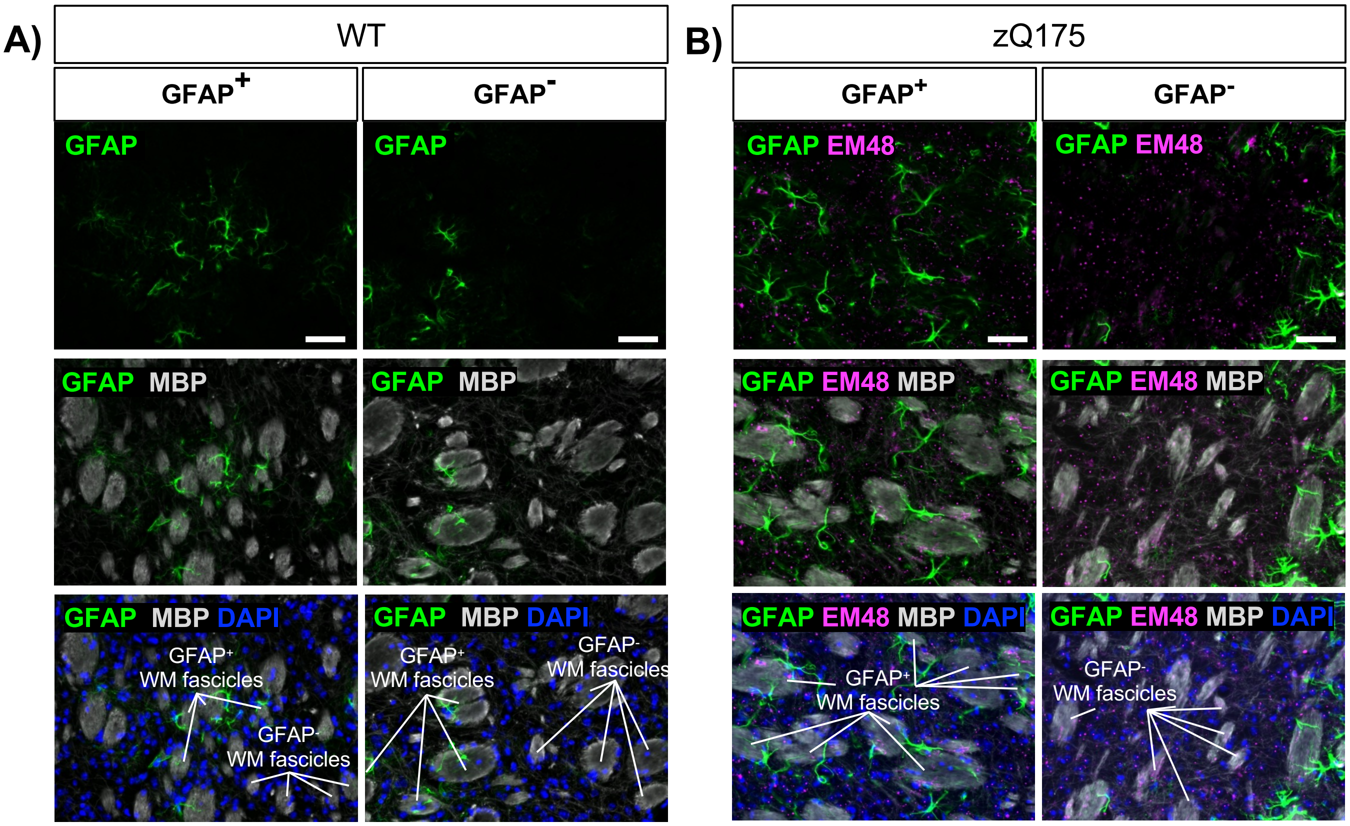

A)
WT
GFAP+
GFAP-
GFAP
GFAP MBP
GFAP MBP DAPI
GFAP+ WM fascicles
GFAP- WM fascicles
B)
zQ175
GFAP+
GFAP-
GFAP EM48
GFAP EM48 MBP
GFAP EM48 MBP DAPI
GFAP+ WM fascicles
GFAP- WM fascicles

Figure S7

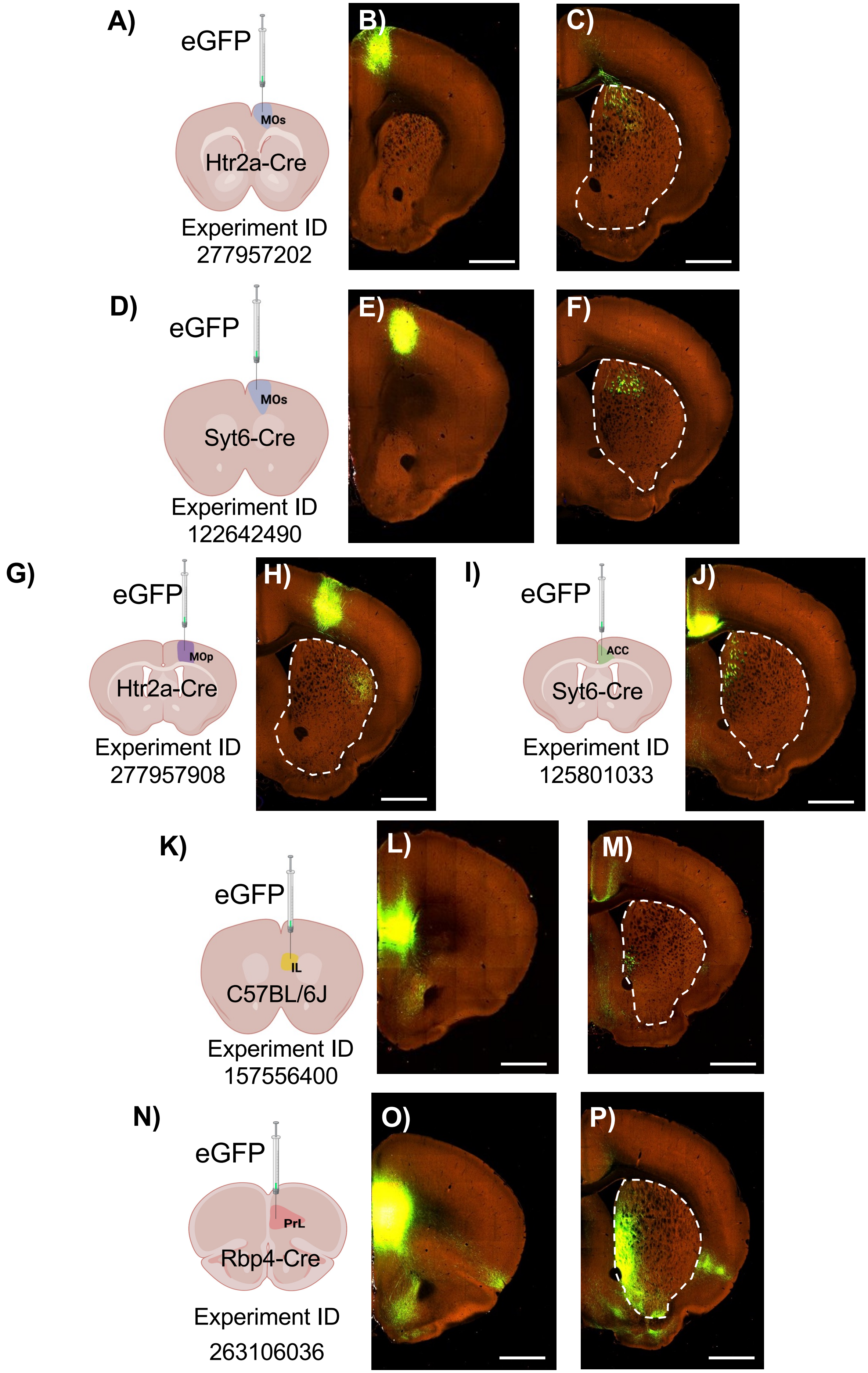

A)
eGFP
MOs
Htr2a-Cre
Experiment ID
277957202
B)
C)
D)
eGFP
MOs
Syt6-Cre
Experiment ID
122642490
E)
F)
G)
eGFP
MOp
Htr2a-Cre
Experiment ID
277957908
H)
I)
eGFP
ACC
Syt6-Cre
Experiment ID
125801033
J)
K)
eGFP
IL
C57BL/6J
Experiment ID
157556400
L)
M)
N)
eGFP
PrL
Rbp4-Cre
Experiment ID
263106036
O)
P)

Figure S8
